# Cytochrome “nanowires” are physically limited to sub-picoamp currents that suffice for cellular respiration

**DOI:** 10.3389/fchem.2025.1549441

**Published:** 2025-03-12

**Authors:** Matthew J. Guberman-Pfeffer, Caleb L. Herron

**Affiliations:** Department of Chemistry and Biochemistry, Baylor University, Waco, TX, United States

**Keywords:** BioDC, multiheme, cytochrome, redox, conductivity, nanowire, geobacter, polarizability

## Abstract

Mineral-respiring microorganisms from hydrothermal vents to terrestrial soils express filaments that electrically connect intracellular respiration to extracellular geochemistry. Filaments dubbed “cytochrome nanowires” (CNs) have been resolved by CryoEM, but whether they are the two-decades-long sought-after physiological “nanowires” remains unproven. To assess their functional competence, we analyzed biological redox conduction in all CNs by computing driving forces in the presence of redox anti-cooperativities, reorganization energies with electronic polarizability, and Marcus rates for diffusive and protein-limited flux models. The chain of heme cofactors in any CN must be densely packed to realize weak (≤0.01 eV) electronic coupling for electron transfer, as evidenced by a single Soret band produced from coincidental absorptions on multiple hemes. Dense packing, in turn, has three consequences: (1) limited driving forces (≤|0.3| eV) due to shared electrostatic microenvironments, (2) strong (≤0.12 eV) redox anti-cooperativities that would accentuate the free energy landscape if the linear heme arrangement did not dictate a contra-thermodynamic oxidation order, and (3) an entropic penalty that is offset by thioether ‘tethers’ of the hemes to the protein backbone. These linkages physically necessitate the rate-throttling T-stacked motif (10-fold slower than the other highly conserved slip-stacked motif). If the sequence of slip- and T-stacked hemes in the CNs had the fastest known nanosecond rates at every step, a micron-long filament would carry a diffusive 0.02 pA current at a physiological 0.1 V, or a protein-limited current of 0.2 pA. Actual CNs have sub-optimal (≤10^2^-fold lower), but sufficient conductivities for cellular respiration, with at most thousands of filaments needed for total cellular metabolic flux. Reported conductivities once used to argue for metallic-like pili against the cytochrome hypothesis and now attributed to CNs remain inconsistent by 10^2^–10^5^-fold with the physical constraints on biological redox conduction through multiheme architectures.

## 1 Introduction

Prokaryotes from hydrothermal vents to terrestrial soils exhale ∼10^6^ electrons/s/cell (∼100 fA/cell) (McLean et al., 2010; Jiang et al., 2013; Gross and El-Naggar, 2015; Lampa-Pastirk et al., 2016; Chabert et al., 2020; Scarabotti et al., 2021; Karamash et al., 2022) through filaments (Reguera, 2018; Baquero et al., 2023) that electrify microbial communities (Lovley, 2017) and biotic-abiotic interfaces (Nealson and Saffarini, 1994). This ‘rock breathing’ strategy for anaerobic life, known as extracellular electron transfer (EET) is ancient (Vargas et al., 1998), ubiquitous (Zhao et al., 2021; Lovley and Holmes, 2022), environmentally significant (Jelen et al., 2016; Jiang Y. et al., 2019; Gu et al., 2021; Zhang et al., 2021), and holds promise for sustainable technologies (Sanjuan-Alberte et al., 2018; Sun et al., 2018; Zhang et al., 2020; Bird et al., 2021; Zou et al., 2021; Guberman-Pfeffer et al., 2024), but only if its mechanistic underpinnings are elucidated.

Filaments from mineral-respiring microorganisms have recently been resolved by cryogenic electron microscopy (CryoEM) to be polymerized multiheme cytochromes (Filman et al., 2019; Wang et al., 2019; Wang et al., 2022b; Baquero et al., 2023; Gu et al., 2023). Though the filaments are dubbed “cytochrome nanowires” (CNs), their physiological role as “nanowires” has not been demonstrated. No experiment can currently measure reliably how well a filament of known identity conducts electrons between molecular redox partners under fully hydrated, physiological conditions (Baquero et al., 2023). But from a theoretical vantage point, can the theory of biological electron transfer [i.e., redox conduction (Boyd et al., 2015)] connect the atomic structures of CNs to their proposed physiological role as mesoscopic electrical conductors?

**FIGURE 1:**
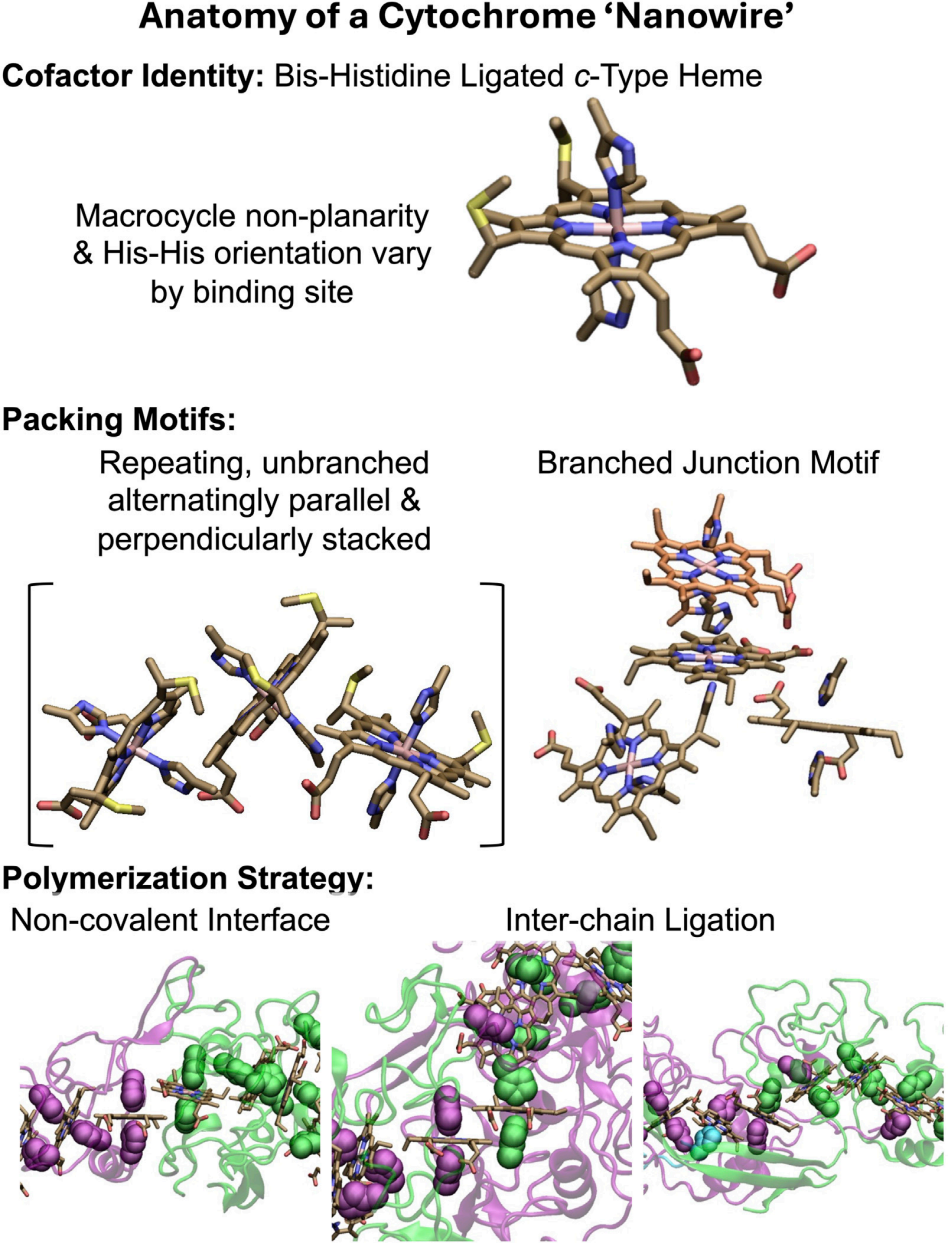
Anatomy of a cytochrome “nanowire.” The heme cofactor, its packing motifs, and filament polymerization strategies are shown. Purple, green, and cyan indicate different protein chains. His ligands are shown in vdW representation. A heme that is coordinated by two differently colored His ligands receives one of them from the adjacent protein chain. The subunit interfaces shown at the bottom from left to right come from OmcZ (PDB 7LQ5) (Gu et al., 2023), OmcS (PDB 6EF8) (Wang et al., 2019), and F2KMU8 (PDB 8E5G) (Baquero et al., 2023). The figure was designed with VMD version 1.9.4a57 (Humphrey et al., 1996).

All structurally characterized CNs (Filman et al., 2019; Wang et al., 2019; Wang et al., 2022b; Baquero et al., 2023; Gu et al., 2023) have the basic anatomy summarized in Figure 1. A single type of cofactor—a bis-histidine ligated *c*-type heme—is repeated hundreds of times and stacked in highly conserved geometries (Iverson et al., 1998; Baquero et al., 2023) to form a micron-long (linear or branched) spiraling chain, which is encased by a mostly unstructured (≥50% turns and loops) protein sheath. Each filament is constructed of a different Lego-like multiheme cytochrome that polymerizes either exclusively through non-covalent interactions, or one or more coordination bonds between protomers.

The filaments are homopolymers of a tetra-, hexa-, or octaheme protein. The filamentous outer-membrane cytochrome (Omc) types E, S, and Z from *Geobacter sulfurreducens* are respectively composed of these different types of multiheme building blocks. Two other tetraheme-based filaments come from *Pyrobaculum calidifontis* and *Archaeoglobus veneficus*. Because the latter proteins have not yet been given convenient common names, they will be referred to by the UniProtKB accession codes A3MW92 and F2KMU8, respectively.

The stacking of hemes to form a chain, and the association of proteins to extend that chain is an architectural design found elsewhere in nature: Chains of 4 (Matias et al., 1997; Taylor et al., 1999), 5 (Moser et al., 2000), 12 (Pokkuluri et al., 2011), 16 (Santos-Silva et al., 2007), 20 (Edwards et al., 2020b), 30 (Dietl et al., 2019), and 192 (Akram et al., 2019) hemes are formed through non-covalent association (Einsle et al., 1999; Rodrigues et al., 2006; Dietl et al., 2019; Edwards et al., 2020b), coordination chemistry (Matias et al., 1997), or genetic grafting and pruning (Pokkuluri et al., 2011; Soares et al., 2022) of other multiheme modules. CNs, however, are unique in that multiheme proteins with two distinct binding interfaces are iteratively paired to form, in principle, an infinitely extendable, cylindrical assembly. That these assemblies extend for μ m-lengths beyond the safety of the outer-membrane and contain ∼10^3^ cofactors, each with a metal ion (iron) of limited bioavailability, is remarkable from a biosynthetic cost-benefit perspective.

But the benefits to a microorganism from expressing CNs remain unclear. Why, for example, is a single bacterium, *G. sulfurreducens*, capable of producing three of the five structurally characterized CNs? Is it because the heme chain common to all CNs have different conductivities that match different respiratory rates, or does the non-homologous protein ‘packaging’ provide environmentally-customized interfaces (Guberman-Pfeffer, 2023b)?

The CryoEM structures have been assumed to explain the molecular underpinnings of EET (Baquero et al., 2023), but biochemical data have also been interpreted as implicating an altogether different filament of so far hypothetical structure (Lovley and Walker, 2019). Solid-state electrical measurements have been assumed to be biologically relevant (Wang et al., 2019), even though the theory of biological electron transfer cannot explain them (Eshel et al., 2020; Guberman-Pfeffer, 2022)—an observation that once was used to argue that the filaments are not cytochromes (Malvankar and Lovley, 2012; Malvankar et al., 2012).

Under pressure to validate the solid-state experiments, theoretical models have proposed biologically implausible electronic coherences over 20 nm (Eshel et al., 2020), predicted spuriously negative redox potentials and a 0.4 V hysteresis (Dahl et al., 2022; Guberman-Pfeffer, 2022) that was even known beforehand not to exist (O’Brien, 2020; Shipps, 2022), or have modeled biologically irrelevant electron transport (as opposed to transfer) through a molecular junction (Livernois and Anantram, 2023).

None of these studies have answered the question: Are the CNs resolved by CryoEM *competent* to discharge the metabolic flux of electrons from a microbial cell? Guberman-Pfeffer recently proposed that a *minimum* of seven filaments would suffice for cellular respiration given the conductivity of a generic heme chain (Guberman-Pfeffer, 2024). How does this prediction hold up to computations on the actual structures?

Herein, we characterize the energetics and kinetics in all structurally resolved CNs, compare their conductivities to the needs of cellular respiration, and deduce physical constraints on the conductivity of any biological heme chain. In so doing, we demonstrate that the multiheme architecture physically limits CNs to sub-optimal but sufficient micro-to-millisiemens conductivities for cellular respiration by EET. If the CNs were the only route for expelling electrons, which is *not* the case, tens of the tetrahemes, hundreds of the hexaheme, or thousands of the octaheme filaments would be needed.

## 2 Methods

All quantum chemical calculations were performed with the Gaussian 16 Rev. A.03 program (Frisch et al., 2016). All other calculations were performed with Python packages available on GitHub that were written by the authors. These packages include: (1) BioDC V2.2 (https://github.com/Mag14011/BioDC/releases/tag/V2.2): A program to assess redox potentials, cooperativities, and conductivity in multiheme proteins; (2) ETAnalysis V1.0 (https://github.com/Mag14011/ETAnalysis/releases/tag/V1.0): A program to compute standard Marcus theory rates, charge diffusion constants, and protein-limited, steady-state multi-particle electron fluxes; (3) EFieldAnalysis V1.0 (https://github.com/Mag14011/ElectricFieldAnalysis/releases/tag/V1.0): A program to compute the electric field at multiple probe atoms in a protein over the course of molecular dynamics (MD) trajectories; and (4) PolReorg V1.0 (https://github.com/Mag14011/PolReorg/releases/tag/V1.0): A program to compute the effect of active site polarizability on outer-sphere reorganization energy for electron transfer.

### 2.1 Structure selection and preparation

Presently available entries in the Protein Data Bank (PDB) for CNs were prepared with BioDC. These include PDB ID 7TFS (OmcE), 8E5F (A3MW92), 8E5G (F2KMU8), 6EF8 and 6NEF (OmcS), and 7LQ5 and 8D9M (OmcZ). BioDC automatically detects the type (*b*/*c*) and axial ligation (His-His/His-Met) of each heme and assigns AMBER FF10 forcefield parameters for the chosen (oxidized/reduced) charge state. BioDC can prepare each structure for constant pH molecular dynamics simulations (including for the propionic acid heme substituents) if desired. For computational tractability when considering all structurally characterized CNs as in the present work, standard protonation states were assigned to each filament. The conclusions were found to be robust to this approximation based on available experimental and computational data (Section 3.2.1).The BioDC program has an interactive interface that writes the selected settings to a file that can be used as an input file for reproducibility. Those files for the present analysis are provided in the GitHub repository.

All the CryoEM structures were resolved in the fully oxidized state and were either used directly after minimization in electrostatic calculations, as done previously (Jiang et al., 2020), or as the starting point for previously published molecular dynamics (MD) simulations in different redox microstates (Guberman-Pfeffer, 2023b) that are re-analyzed in the present work. The goal is to *test*, not to *assume*, the competence of these resolved structures for their proposed physiological role.

A comprehensive set of experimental structural data for a multiheme cytochrome is impractical or even technically impossible given the coupled redox, protonation, and conformational equilibria in these systems. For a protein with n redox- and m pH-active residues, there are 2^n+m^ possible microstates, because each center can be either oxidized/reduced or deprotonated/protonated. A tetra-, hexa-, or octaheme protein with just one pH-active residue would have 32, 128, and 512 microstates, respectively. In the actual CNs, pH-active residues include, *per subunit,* 8–16 propionic acid groups, 9–15 aspartic acids, 3–13 glutamic acids, 7–14 lysines, 1–5 histidines, and 1–22 tyrosines. Theory and computation are the *only* practical means to study, at a mechanistic level with atomistic resolution, the enormous set of microstates populated by a multiheme cytochrome. The lack of experimental structural data is an argument for, not against, the use of computations to obtain physical insights (Popović et al., 2001).

MD simulations at constant pH and solution potential can, in principle, provide the needed structural ensembles (Cruzeiro et al., 2020), but the computational cost is prohibitive, especially for comparing multiple CNs. To date, such simulations have only been applied to a trimeric model of the OmcS filament (Guberman-Pfeffer, 2022).

A more approximate but extensively employed approach involves Monte Carlo sampling of redox and protonation states in continuum electrostatic calculations on a structure that is mostly (i.e., rigid backbone) or entirely fixed at experimental coordinates in a specific oxidation state (Mao et al., 2003; Popović and Stuchebrukhov, 2004; Zheng and Gunner, 2009). Protein and solvent responses to charge changes in this approach are implicitly encoded in the chosen dielectric constant. An internal dielectric of 2 reflects electronic polarization, whereas larger values implicitly capture larger (nuclear) reorganization effects. In all electrostatic calculations presented here, the internal dielectric constant was 5.2–7.6, as computed by Equation 3 below.

Other theoretical studies on CNs to date have performed MD sampling in select microstates (Dahl et al., 2022; Guberman-Pfeffer, 2023b), or assumed that redox-linked conformational transitions can be neglected altogether (Eshel et al., 2020; Jiang et al., 2020; Livernois and Anantram, 2023). The OmcS protein was simulated in multiple redox states, each for tens to hundreds of nanoseconds (Dahl et al., 2022; Guberman-Pfeffer, 2023b) using the oxidized CryoEM structure as a starting point. These simulations included states in which all hemes were oxidized, all hemes were reduced, each heme was separately reduced while all other hemes were oxidized (6 different states), and each heme was separately oxidized while all other hemes were reduced (6 different states). None of these simulations revealed large-scale conformational changes in protein structure. The conclusions regarding the energetics and kinetics of electron transfer at 300–310 K from these simulations were well reproduced at much less computational expense by a study that assumed the rigid, fully-oxidized CryoEM geometry after energy minimization for all microstates (Jiang et al., 2020).

The single-structure approximation is clearly imperfect, but justified by its established precedents for yielding physically meaningful insights (Soares and Baptista, 2012), the small refinements to electron transfer driving forces in the CNs found from dynamical simulations to date, the implicit modeling of some conformational responses with internal protein dielectric constants >2 in the presented electrostatic calculations, the consideration of multiple CryoEM structures where available, and the intractability of more sophisticated computational protocols at present. With the exception of driving forces, all other reported energetic quantities are based on MD simulations equilibrated in different redox microstates.

### 2.2 Redox potentials, driving forces, and redox cooperativities

BioDC was used to estimate the oxidation energy of heme *i* while all other hemes were in the reduced state (Equation 1); and the change in the oxidation energy of heme *i* due to the oxidation of heme *j*, while all other hemes were in the reduced state (Equation 2).

$$\Delta E_{i}=\left(E_{i_{\text{ox}}}-E_{i_{\text{red}}}\right)$$ (1)

$$\Delta \Delta E_{i,j}=\left(E_{i_{\text{ox}},j_{\text{ox}}}-E_{i_{\text{red}},j_{\text{ox}}}\right)-\left(E_{i_{\text{ox}},j_{\text{red}}}-E_{i_{\text{red}},j_{\text{red}}}\right)$$ (2)

A more negative (positive) $\Delta E_{i}$ indicates that the oxidized state is relatively stabilized (destabilized) and correlates with a more negative (positive) redox potential if the electrostatic contribution dominates the free energy of oxidation. In the context of electron transfer, the driving force is related to the difference in Δ Ei between donating and accepting groups. Because those groups are chemically identical in the CNs, non-electrostatic contributions are expected to largely cancel.

Equation 1 was used previously to estimate driving forces in OmcS (Jiang et al., 2020); Equation 2 is an extension of the idea to evaluate heme redox cooperativities as defined in (Turner et al., 1996).

Consistent with this approach, electrostatic interactions were shown to dominate the redox potentials in Omc- E, S, and Z (Guberman-Pfeffer, 2023b). Furthermore, the implementation of Equations 1 and 2 successfully reproduced the variation of redox potentials for hemes, with and without anti-cooperative interactions, in aqueous and membranous environments (Hardy et al., 2024). As another benchmark, the experimental site and site-site interaction energies for the triheme Periplasmic protein cytochrome A from *G. sulfurreducens* were reproduced with maximum deviations of ≤0.05 and ≤0.01 eV, respectively (Supplementary Figure 1).

To evaluate each energy term, BioDC interfaces with the Poisson-Boltzmann Solvation Area (PBSA) model of the AmberTools suite (Case et al., 2023). The external dielectric constant was set to 78.2 for a bulk aqueous environment at 25°C and the implicit salt concentration was set to a physiologically relevant 0.15 M.

The internal dielectric constant of the protein was estimated from the total solvent accessible surface area (SASA) for each donor-acceptor pair by Equation 3,

$$\varepsilon_{s}=\alpha +\beta \left(\text{SAS}A_{D}+\text{SAS}A_{A}\right)$$ (3)

where α=5.18 and $\beta =0.016{\;Å}^{-2}$ . These parameters were previously developed (Breuer et al., 2014) so that the Marcus continuum equation (Equation 4 below) reproduces reorganization energies from MD simulations performed with a polarizable forcefield.

When computing heme redox anti-cooperativities by Equation 2, the estimation of the interior dielectric constant was simplified in view of the fact that calculations were needed for every possible pair of hemes (not just adjacent donor-acceptor pairs). The internal dielectric constant was set for all heme pairs as the average of the values computed by Equation 3 for adjacent heme pairs.

### 2.3 Reorganization energy

#### 2.3.1 Empirically parameterized Marcus Continuum approach

BioDC estimates the outer-sphere reorganization energy $\left(\lambda_{\text{out}}\right)$ by assuming Δq units of charge are transferred between spherical donor and acceptor groups (radii $R_{D}$ and $R_{A}$ ) at an R center-to-center distance in a bulk medium characterized by static $\left(\varepsilon_{s}\right)$ and optical $\left(\varepsilon_{\text{opt}}\right)$ dielectric constants. The expression, Equation 4, is known as the Marcus continuum equation.

$$\lambda_{\text{out}}=\Delta q^{2}\left(\frac{1}{\varepsilon_{\text{opt}}}-\frac{1}{\varepsilon_{s}}\right)\left(\frac{1}{2R_{D}}+\frac{1}{2R_{A}}-\frac{1}{R_{\text{DA}}}\right)\times 27.2114$$ (4)

Equation 4 was shown before (Breuer et al., 2014) to well reproduce $\lambda_{\text{out}}$ from all-atom MD simulations conducted with an electronically polarizable forcefield if $R_{D}=R_{A}=4.6\;Å$ for an effective heme radius, $\varepsilon_{\text{opt}}=1.84$ , and $\varepsilon_{s}$ is assumed to be a linear function of the total (donor + acceptor) SASA (Equation 3),

#### 2.3.2 Vertical energy distributions and polarizability corrections

The thermally-sampled distributions of instantaneous (vertical) changes in electrostatic interaction energy $\left(\Delta U\right)$ between the donor or acceptor and the rest of the environment upon oxidation/reduction were used in a second approach to compute $\lambda_{\text{out}}$ .

If ΔU for the reactant (R) and product (P) states is Gaussian distributed and ergodically sampled according to Boltzmann statistics on the timescale of the electron transfer, $\lambda_{\text{out}}$ is equivalently defined in terms of the average $\left({\langle \Delta U\rangle }_{x},x=R\text{ or }P\right)$ ; Equation 5 or variance $\left(\sigma_{x}\right)$ ; Equation 6 of the vertical energy distributions in the two states. These definitions are respectively known as the Stokes-shift $\left(\lambda^{\text{st}}\right)$ and variance $\left(\lambda^{\text{var}}\right)$ reorganization energies; if they are not equivalent, the reaction reorganization energy $\left(\lambda^{\text{rxn}}\right)$ that should be used in Marcus theory is defined in terms of their ratio (Equation 7) (Martin et al., 2019). In Equations 5–7, $k_{b}$ and T are respectively the Boltzmann constant and absolute temperature.

$$\lambda^{\text{st}}=\frac{1}{2}\left({\langle \Delta U\rangle }_{R}-{\langle \Delta U\rangle }_{P}\right)$$ (5)

$$\lambda^{\text{var},R}=\frac{\sigma_{R}}{2k_{b}T};{\;\lambda }^{\text{var},P}=\frac{\sigma_{P}}{2k_{b}T}$$ (6)

$$\lambda^{\text{rxn}}=\frac{{\left(\lambda^{\text{st}}\right)}^{2}}{\left(\sigma_{R}+\sigma_{P}\right)/2}$$ (7)

Because the inner-sphere contribution is much less than the outer-sphere contribution for heme groups, $\lambda^{\text{rxn}}\approx \lambda_{\text{out}}$ in this work. However, it will be noted in the results when $\lambda^{\text{rxn}}$ is different from the expectations of standard Marcus theory because $\lambda^{\text{st}}\neq \lambda^{\text{var},R/P}$ .

##### 2.3.2.1 Coulombic vertical energies

ΔU is typically computed as the instantaneous Coulombic energy change $\left(\Delta U_{\text{coul}}\right)$ for the donor-acceptor pair in the R or P state from classical MD simulations conducted using non-polarizable forcefields. $\Delta U_{\text{coul}}$ is given by the product of the change in partial atomic charge for each atom of the donor and acceptor $\left(\Delta q\right)$ with the electrostatic potential $\left(\phi \right)$ exerted by the environment on that atom, summed over all the atoms of the two groups (Equation 8).

$$\Delta U_{\text{coul}}=\sum_{i}\Delta q_{i}\phi_{i}$$ (8)

$\Delta U_{\text{coul}}$ was computed with the CPPTRAJ program (Roe and Cheatham III, 2013) of the AmberTools suite; an example CPPTRAJ input script is provided with the PolReorg package. Plots showing the distributions of $\Delta U_{\text{coul}}$ in the R and P states, as well as the convergence of $\lambda^{\text{st}}$ , $\lambda^{\text{var},R}$ , and $\lambda^{\text{var},P}$ , are shown for each electron transfer in Omc- E, S, and Z in Supplementary Figures 2–4.

##### 2.3.2.2 Perturbative correction for active site polarizability

ΔU was alternatively expressed for the R or P state by perturbation theory (Equation 9) as the sum of a reference energy $\left(\Delta U_{0}\right)$ , the Coulombic term $\left(\Delta U_{\text{Coul}}\;\right)$ ; Equation 8 and the polarization energy $\left(\Delta U_{\text{pol}}\right)$ ; Equation 10 (Martin et al., 2019).

$$\Delta U\approx \Delta U_{0}+\Delta U_{\text{coul}}+\Delta U_{\text{pol}}$$ (9)

$\Delta U_{\text{pol}}$ for the donor or acceptor is related to the difference in the second-rank polarizability tensor between the oxidation states for that group $\left(\Delta \alpha \right)$ and the electric field vector $\left(E\right)$ acting on it (Equation 10), which is approximated according to literature precedent (Matyushov, 2011; Dinpajooh et al., 2016) (although over-simplistically) as the field acting on the Fe center of each heme. Note that because Δ always means final–initial, $\Delta \alpha =n\left(\alpha^{\text{ox}}-\alpha^{\text{red}}\right)$ , where n=1 for the donor and −1 for the acceptor.

$$\Delta U_{\text{pol}}=-\frac{1}{2}\sum_{j}\sum_{k}\Delta \alpha_{\text{jk}}E_{j}E_{k}$$ (10)

To evaluate Equation 10, Δα was computed with a variety of model chemistries for a His-His ligated heme group in both vacuum and configurations of the distinct binding sites of the OmcS filament sampled during MD simulations. To facilitate a comparison with prior work, Δα was also computed for a His-Met ligated heme group in vacuum and configurations of cytochrome *c* from MD simulations. The benchmarking data is in Supplementary Tables 1 and 2.

The electric field at the heme-Fe centers was computed over the course of MD trajectories by the EFieldAnalysis program. EFieldAnalysis implements the methodology for a probe atom found in the TUPÂ package (Polêto and Lemkul, 2022), but unlike TUPÂ, EFieldAnalysis wraps molecules instead of atoms at the periodic boundary to preserve molecular integrity. It also parallelizes the calculation of the total field vector over multiple CPUs for a more efficient assessment of long trajectories. Equations 9 were implemented in the PolReorg python package.

### 2.4 Electron transfer rates, charge diffusion constants, and steady-state fluxes

Non-adiabatic Marcus theory electron transfer rates were computed with BioDC and used with (1) the analytical Derrida equation to obtain a charge diffusion constant (Derrida, 1983), and (2) a multi-particle steady-state kinetics model to obtain the protein-limited electron flux (Breuer et al., 2014; Jiang et al., 2017; Jiang et al., 2020). These two kinetic models are implemented in the ETAnalysis program with code graciously contributed by Fredrik Jansson (Derrida module) and Jochen Blumberger and Xiuyun Jiang (flux module). The latter module was modified to read the energy matrix of site and site-site interaction energies calculated by BioDC. With that information, it self-consistently updates the redox potentials by weighing the interactions according to the electron occupancies. The self-consistent procedure was improved by implementing an adaptive mixing scheme to aid convergence.

### 2.5 Monte Carlo sampling of energetic parameters for target charge diffusion constants

The Parameter Explorer module of ETAnalysis was used to sample sets of electronic couplings, reaction free energies, and reorganization energies for a specified sequence of slip- and T-stacked heme pairs. A set is accepted if it gives a desired charge diffusion constant within a specified tolerance (here ±10%). A KD-tree algorithm was used to remove duplicate sets of parameters.

### 2.6 UV-vis spectral analysis

#### 2.6.1 Structure preparation

The model of a heme cofactor shown in Supplementary Figure 5 was prepared by (1) cleaving and capping with hydrogens the C_β_-C_α_ bonds that connect the cofactor to the protein via axial His ligands and thioether bonds with Cys residues; (2) removing the propionic acid groups and treating them as part of the electrostatic environment (Jiang et al., 2019b; Nattino et al., 2019), and (3) for some computations, replacing methyl substituents with hydrogen atoms. The latter truncation had a minimal spectral effect (Supplementary Figure 6).

The heme model was optimized in the closed-shell singlet reduced (formally Fe^2+^) state with the Becke, three-parameter, Lee–Yang–Parr (B3LYP) density functional (Beck, 1993; Stephens et al., 1994) and a mixed basis set comprised of LANL2DZ for the Fe center (Hay and Wadt, 1985) and 6-31G(d) for H, C, N and S (Hariharan and Pople, 1973)). A local minimum was found, as confirmed by a harmonic vibrational analysis (i.e., no negative frequencies). Focus was given to the reduced state to avoid concerns over spin contamination in the open-shell doublet oxidized state, and to make converging the supramolecular hexa-heme calculation for excitonic effects described below more tractable. However, prior work has indicated that similar theoretical methods correctly capture the spectral difference between the reduced and oxidized states (Neu et al., 2022).

#### 2.6.2 Electrostatic contribution to spectral tuning

The unmethylated heme model was superimposed on and replaced each of the six hemes in the central subunit of an OmcS trimer. The superpositions were performed on 216–300 configurations of the protein (sampling rate = 1 frame/ns) generated by previously published MD simulations (Guberman-Pfeffer, 2023b) in which the target heme was reduced while all other hemes were oxidized. This procedure leveraged the semi-rigid nature of the heme group to preserve the influence of the environment while avoiding the unreliability of MD-generated geometries in quantum mechanical calculations, as well as the intractability of 10^3^ quantum mechanical/molecular mechanical optimizations or dynamical simulations.

Time-dependent (TD)-DFT calculations were performed on these configurations with the superimposed hemes in the QM region and all other atoms converted to an electrostatic background. The CAM-B3LYP functional is known to produce a blue-shifted absorption spectrum for heme (Graves et al., 2016), and B3LYP does the same (Supplementary Figure 6). On the hypothesis that the blue-shifting results from too much electron localization, a functional with a smaller fraction of Hartree-Fock exchange (specifically BLYP) was tested and found to give closer agreement with the expected position of the Soret absorption maximum (∼420 nm) for a reduced heme (Supplementary Figure 6). The same mixed basis set used for geometry optimization was used for the spectral simulations.

#### 2.6.3 Conformational contribution to spectral tuning

The heme cofactor was displaced in 0.1 Å increments from 0.0 to 1.0 Å along each of the ruffling and saddling deformation normal coordinates (Kingsbury and Senge, 2021) and the absorption spectrum was simulated for each conformer. The displaced geometries were prepared with the Normal Coordinate Structure program graciously provided by Christopher J. Kingsbury (Kingsbury and Senge, 2021). The structures were optimized with all dihedrals fixed before simulating the spectra with TD-DFT.

#### 2.6.4 Excitonic contribution to spectral tuning

The Frankel exciton model (Curutchet and Mennucci, 2005; Russo et al., 2007) implemented in Gaussian 16 Rev. A.03 was used to compute the UV-vis spectrum and to quantify excitonic couplings. For this calculation, the heme with methyl substituents was superimposed on the hemes in a configuration of the protein generated by MD and each heme was defined as a separate QM region. The protein-water environment was again included as an electrostatic background.

The calculation was performed with CIS and a mixed (LANL2DZ for Fe; 6-31G(d) for H, C, N, and S) basis set, as has been done previously (Guberman-Pfeffer and Gascón, 2018) to avoid the deficiencies of TD-DFT (Magyar and Tretiak, 2007).

## 3 Results and discussion

Biological electron transfer in multiheme proteins is generally pictured in terms of a succession of reduction-oxidation (redox) reactions in which the electron takes multiple, incoherent “hops” from site-to-site (Blumberger, 2018). In this view, an electron resides on a heme group until an infrequent thermal fluctuation overcomes the energy barrier for the electron to be on the adjacent heme, at which time the electron ‘hops’ to that heme with a probability less than unity (i.e., non-adiabatically).

The activating thermal fluctuation is rare because the energy barrier is usually several multiples of the available thermal energy. In the time spent waiting for the energetically propitious configuration to be realized, the electron transferred in the prior ‘hop’ is thermally equilibrated and loses coherence. Once the barrier for the next ‘hop’ is overcome, the probability for transferring the electron is small, because only the tails of the wavefunctions on adjacent hemes overlap in the intervening space due to the exponential decay of the wavefunctions with distance from the edge of the macrocycle.

The rate constant for each electron transfer $\left(k_{\text{et}}\right)$ is therefore the product of probabilities for thermal activation $\left(\rho \right)$ and electronic tunneling $\left(\kappa \right)$ which, in the high temperature (classical) limit, takes the form of standard Marcus theory (Marcus, 1956; Marcus, 1960):

$$k_{\text{et}}=\text{κ}\text{ρ}=\frac{2\pi \langle H^{2}\rangle }{\hbar }\frac{e^{\frac{-{\left(\Delta G^{\circ }+\lambda^{\text{rxn}}\right)}^{2}}{4\lambda^{\text{rxn}}k_{b}T}}}{\sqrt{\left(4\pi \lambda^{\text{rxn}}k_{b}T\right)}}$$ (11)

Apart from fundamental constants ( ℏ = reduced Plank constant; $k_{b}$ = Boltzmann constant), $k_{\text{et}}$ depends on three energetic parameters: (1) the thermally averaged electronic coupling squared $\left(\langle H^{2}\rangle \right)$ , which describes the overlap of wavefunctions on the donor and acceptor, (2) the reaction reorganization energy (λ^rxn^), which describes the energy penalty for rearranging nuclei to be consistent with the product state before the electron is transferred, and (3) the reaction free energy $\left(\Delta G^{\circ }\right)$ , which is given by the redox potential difference between the charge donor and acceptor. The term $\frac{{\left(\Delta G^{\circ }+\lambda^{\text{rxn}}\right)}^{2}}{4\lambda }$ is defined as the activation energy $\left(E_{a}\right)$ .

Each of these energetic terms is analyzed in turn for all structurally characterized CNs (Sections 3.1–3.4). The associated rates (Section 3.5) are assembled into diffusive and steady-state kinetic models of long-range electron transfer (Section 3.6). The computed conductivities are compared to solid-state electrical measurements and the demands of cellular respiration (Section 3.6).

### 3.1 Electronic versus excitonic coupling

Electronic coupling strengths for the heme packing geometries present in CNs have extensively and consistently been found to be sub-thermal energy at 300 K (Blumberger, 2018). And yet, UV-vis spectra for multiheme proteins were recently interpreted as suggesting an electronic band structure (van Wonderen et al., 2024). This section clarifies that quantum mechanical electronic couplings in CNs are weak, whereas excitonic couplings are almost entirely classical and 10-fold larger. The excitonic couplings give insight into multiheme redox anti-cooperativities, which are important later.

#### 3.1.1 Heme-heme electronic coupling strengths are sub-thermal energy

Electronic couplings are principally determined by the distance and orientation of the charge donating and accepting groups (Smith et al., 2006). The electrostatic environment causes only a ∼10% perturbation (Blumberger, 2008; Guberman-Pfeffer, 2022).

**FIGURE 2:**
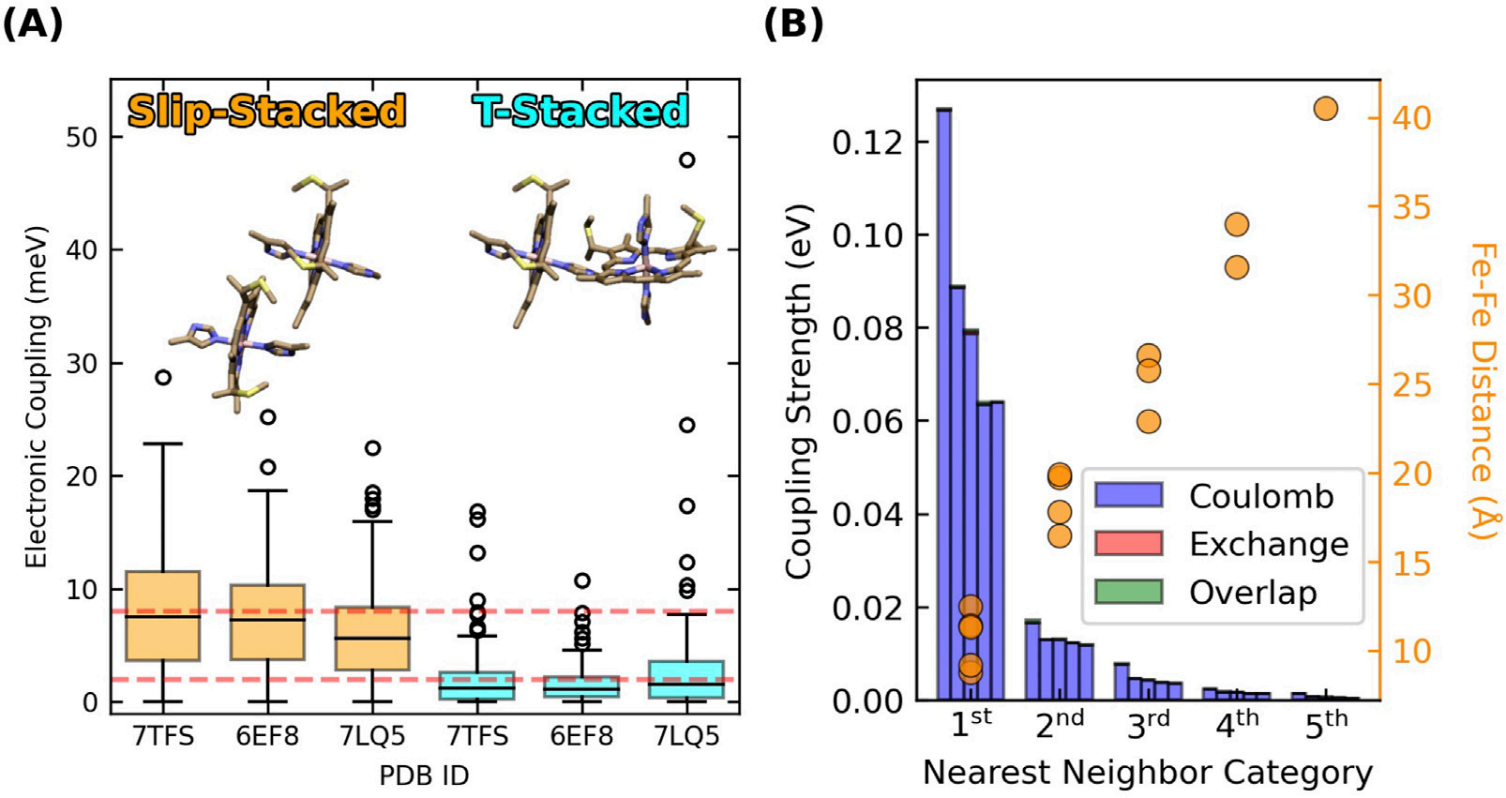
Slip- and T-stacked hemes in the multiheme architecture of cytochrome “nanowires” have weak quantum mechanical electronic couplings but stronger classical excitonic interactions. **(A)** Electronic couplings in Omc- E, S, and Z. The data is reproduced from (Guberman-Pfeffer, 2023b). **(B)** Excitonic couplings computed for the hemes in OmcS at the CIS/[LANL2DZ (Fe):6-31G(d)(H,C,N,S)] level of theory.

Adjacent hemes in the known CNs adopt two highly conserved packing geometries found throughout the class of multiheme proteins (Wang et al., 2022a; Wang et al., 2022b), and preserved under hundreds of nanoseconds of MD simulations (Guberman-Pfeffer, 2023b). These packing geometries are the parallel-displaced (“slip-stacked”, denoted “S”) and perpendicular (“T-stacked”, denoted “T”) motifs (Figure 2A).

Four of the five CNs (OmcE, A3MW92, F2KMU8, and OmcS) exclusively have a (TS)_n_ pattern, whereas OmcZ has a (TSSTSTS)_n_ pattern along the main chain, as well as a heme that branches from the main chain.

Root-mean-squared electronic couplings $\left(\sqrt{\langle H^{2}\rangle }\right)$ previously computed for Omc- E, S, and Z were 0.007–0.010 eV for slip-stacked and 0.002–0.006 eV for T-stacked geometries (Figure 2A) (Guberman-Pfeffer, 2023b). These values are in good agreement with other studies on OmcS (Jiang et al., 2020; Dahl et al., 2022) and multiheme cytochromes in general (Blumberger, 2018). Given the conserved geometries and coupling ranges, values of 0.008 and 0.002 eV for slip- and T-stacked heme pairs (red dashed lines in Figure 2A) are adopted to analyze the electron flux through all CNs in the present work.

#### 3.1.2 Geometric constraints on heme couplings make electron transfers sub-optimal

T-stacked hemes intrinsically have lower electronic couplings compared to slip-stacked hemes (Section 3.1.1). The consequence, according to prior experiments (van Wonderen et al., 2019; van Wonderen et al., 2021) and the computations of Section 3.5 (*vide infra*) is ∼10-fold slower electron transfer rates. If T-stacked hemes are a ‘speed-bump’, why are ∼50% of all heme pairs in CNs of this rate-throttling variety?

To transfer electrons, hemes must be placed within tunneling distance (≤14 Å). The closer the hemes are packed, the more readily endergonic steps can be tolerated and traversed (Moser et al., 2000). Close proximity improves wavefunction overlap for electron transfer, but even for hemes in van der Waals contact, electronic couplings are ≤0.01 eV (Figure 2A).

At the same time, an entropic penalty for organizing densely packed hemes in a protein interior must be offset. Thioether linkages between Cys residues and the hemes are perhaps needed, *inter alia* (Bowman and Bren, 2008), to oppose entropically-driven dissociation (Allen et al., 2003); multiheme proteins with more than three hemes exclusively have *c*- instead of *b*-type hemes (Edwards et al., 2020a), where the difference is the presence of the thioether linkages.

These linkages work in concert with axial ligands donated by other residues to the heme-Fe centers to place geometrical constraints on the packing geometries. Most notably, the slip-stacked geometry that is most favorable for heme-to-heme electron transfer cannot be physically accommodated for more than two consecutive heme pairs by the protein backbone to which the hemes are tethered (Baquero et al., 2023). The geometrically acceptable possibilities include a continuous chain of T-stacked hemes (Baquero et al., 2023), which is not observed, or the typical alternating pattern of parallel and perpendicularly-stacked hemes, as found in the CNs. Either way, the rate-throttling T-stacked motif is *unavoidable* in a cytochrome with more than three *c*-type hemes, and cytochromes with this many hemes only have the *c*-type variety.

Electron transfer through multiheme cytochromes is physically constrained by the geometry of their construction. The implication is that CNs are *not* optimized (in an absolute sense) for long-range electron transfer, nor do they need to be. The slowest experimentally measured (T-stacked) heme-to-heme electron transfer rate of $8.7\times 10^{6}\;s^{-1}$ (van Wonderen et al., 2019) is ∼10^4^-fold larger than either typical intracellular enzymatic turnover (Noy et al., 2006) or the fastest computed interfacial multiheme-to-hematite surface electron transfer rate (Kerisit et al., 2007), Electron ingress and egress in/from CNs is expected to be *much slower* than intra-filament electron transfer. Section 3.6 confirms this expectation for the CryoEM structures based on their computed conductivities and thereby indicates that these structures are competent for a physiological role in cellular respiration.

#### 3.1.3 Multiheme UV-vis spectra misinterpreted as evidence for electronic band structure

If electronic couplings in CNs, and multihemes more generally are weak, how can the appearance of a single Soret band in their UV-vis spectra be understood? Multihemes were proposed to have an electronic band structure to explain why the hemes do not have distinct absorption signatures (van Wonderen et al., 2024). Weakly coupled hemes under physiological conditions, however, cannot support long-range delocalization. What, then, is the origin of the single Soret band, for example, in OmcS (Neu et al., 2022)?

The key quantity to consider is excitonic, as opposed to electronic coupling. Figure 2B shows the five largest heme-heme excitonic couplings between excited states for all possible pairs of hemes in a subunit of OmcS, grouped by their nearest-neighbor relationships (first through fifth).

Heme-heme excitonic couplings are almost entirely Coulombic in nature and exceed electronic couplings by ∼10-fold for the same pairs of adjacent (first nearest neighbors) hemes with Fe-Fe distances of 9–12 Å. Longer range interactions are comparable to thermal energy.

These observations follow from the distinct physical origins of electronic and excitonic couplings: The electronic couplings in Figure 2A require orbital overlap, decay exponentially with distance, and only involve the tails of the donor and acceptor wavefunctions. The excitonic couplings in Figure 2B do not require orbital overlap, involve interactions of the full transition dipole moments on the two hemes, and decay with a slower r^−3^ dependence. Thus, Figure 2 indicates that hemes in CNs are weakly electronically coupled because of poor wavefunction overlap in highly conserved packing geometries, but more strongly excitonically coupled because of Coulombic interactions at short distances.

Though excitonic couplings are stronger than electronic couplings, their presence is still not spectrally resolvable for the hexaheme homopolymer OmcS. The 422-nm Soret band for OmcS has a full-width-at-half-maximum that is ∼36 nm (Neu et al., 2022), only twice the full-width-at-half-maximum for a single heme in solution or bound to a globin (Di Pace et al., 1992; Leone et al., 1994). This observation can be understood by considering the factors that can distinguish the Soret absorptions from multiple hemes.

**FIGURE 3:**
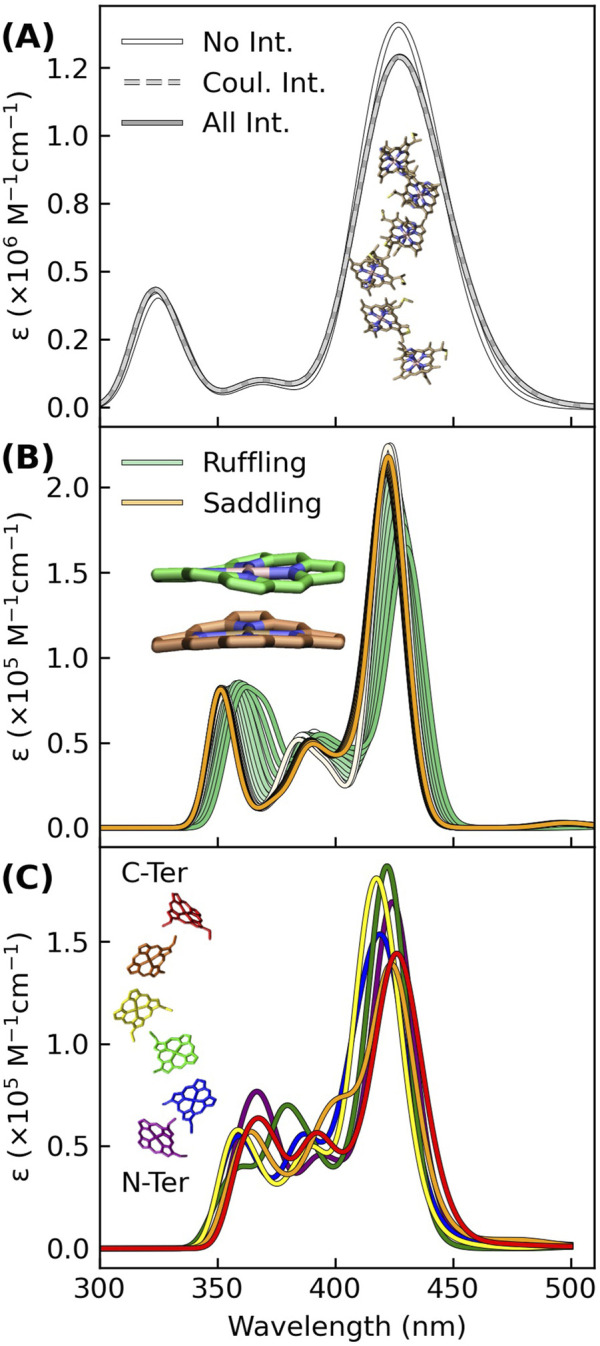
Excitonic, conformational, and electrostatic factors minimally distinguish the spectral signatures of each heme in OmcS, thereby giving a Soret band that is a superposition of nearly coincidental absorptions. **(A)** Spectra simulated for the shown hexaheme array with none, only Coulombic, or all (Coulombic, exchange, and overlap) site-site interactions. **(B)** Spectra simulated for the heme group systematically displaced by 0.1 increments from 0.0 to 1.0 Å along the ruffling and saddling normal coordinate deformations that were previously found to be most populated by the hemes in OmcS (Guberman-Pfeffer, 2023b). **(C)** Thermally averaged spectra of each heme (#1–#6) within the central subunit of an OmcS trimer. **(A–C)** The spectra were simulated using either CIS **(A)** or TD-BLYP **(B, C)** with a mixed basis set [LANL2DZ (Fe):6-31G(d)(H,C,N,S)] and uniformly shifted to align the Soret maximum to the experimental 420 nm position. Gaussians were fit to each transition with a broadening factor of 0.05 eV to simulate the lineshapes.

Figure 3A shows that Gaussian-broadened spectra simulated within a Frankel exciton model are very nearly identical when none, only Coulombic, or all (Coulombic, exchange, and overlap) site-site interactions for the hemes in OmcS are included. Displacements along the two most populated normal coordinate deformations of the heme macrocycles in OmcS (Guberman-Pfeffer, 2023b) only cause an ∼8 nm variation in the Soret absorption maximum (Figure 3B). A similarly small spread is produced by the distinct binding-site electrostatics of the protein-water environment for the different hemes (Figure 3C). Even if these tuning effects are additive, the spread in absorption maxima of the individual hemes would be within the envelope of the overall Soret band.

Taken together, conformational, excitonic, and electrostatic interactions have a fairly minor influence on differentiating the absorption signatures of each heme in OmcS, and presumably other multiheme cytochromes. Instead of all hemes having a single absorption because of electronic delocalization, the separate hemes have nearly coincidental absorptions that are spectrally unresolved. The single Soret band for a multiheme is not inconsistent with weakly electronically coupled hemes.

### 3.2 Redox potentials, anti-cooperativities, and reaction free energies

Only homo, as opposed to heteropolymeric CNs have so far been discovered. Homopolymerization has the interesting consequence that the overall intra-filament driving force is zero: The rise and fall in free energy for an electron through a subunit must repeat when the electron gets to the next subunit.

The driving force between the redox half reactions connected by the CNs is expected to be ∼0.1 V (Bird et al., 2011). Even if the potential falls off linearly across the micron-long filament, the contribution to the energetics at the level of heme-to-heme electron transfers is less than thermal energy (Polizzi et al., 2012). Furthermore, because the electron transfers are between chemically identical bis-histidine-ligated *c*-type hemes, each reaction is a self-exchange and there is formally no driving force.

The heme-to-heme driving forces are not strictly zero, however, as the redox potential of each heme is differently affected by the heterogeneous protein-water environment (Guberman-Pfeffer, 2023b). Two additional electrostatic effects modify these potentials (Fonseca et al., 2012): redox anti-cooperativity, where the oxidation state of each heme influences the oxidation potential of all other hemes, and the redox-Bohr effect, where protonation changes for titratable residues couple to heme-centered redox transitions to produce positive cooperativity. Redox-linked conformational changes can override these electrostatic influences (Fonseca et al., 2012; Barrozo et al., 2018).

The following sub-sections delineate tradeoffs and physical constraints for driving forces that are encoded in the multiheme architecture of CNs; namely: (1) The dense packing of hemes causes electrostatic microenvironments to be shared and redox potential differences between adjacent hemes in van der Waals contact to be small, with or without redox-Bohr interactions; and (2) Dense packing also causes strong (∼0.1 eV) redox anti-cooperativities, but the linear topology of CNs makes the $\Delta G^{\circ }$ landscape largely insensitive to these heme-heme interactions.

#### 3.2.1 Shared electrostatic microenvironments limit driving forces in multihemes

The potential of a redox transition in a multi-center redox protein can be expressed as the sum of site energies and site-site interaction energies, which are respectively represented by the diagonal and off-diagonal elements of an energy matrix (Turner et al., 1996). Higher order (three or more) multi-center interactions are not considered, in accordance with experimental work on multiheme cytochromes (Turner et al., 1996).

**FIGURE 4:**
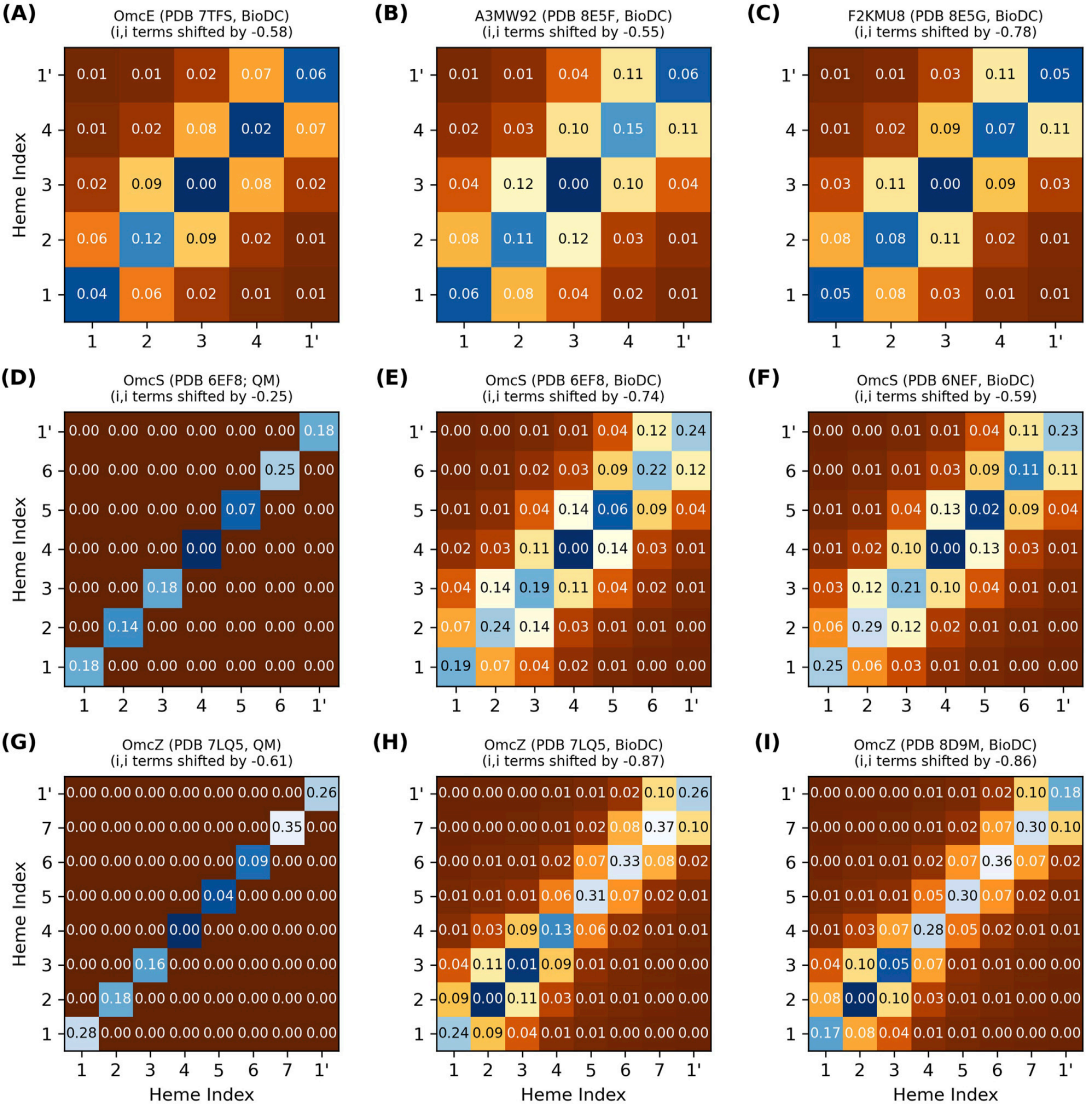
Thermodynamic characterization of relative heme oxidation energies (diagonal elements) and heme-heme interactions energies (off-diagonal elements) in the structurally characterized cytochrome “nanowires.” All energy matrices **(A–I)** were computed with BioDC, except **(D, G)** which are from a prior QM/MM investigation (Guberman-Pfeffer, 2023b) that *assumed* all off-diagonal elements were zero. For each matrix, the hemes are indexed in their linear sequence from 1 to N, and “1’” indicates the first heme of the next subunit. All energies are in eV.

Figure 4 shows the energy matrix computed with BioDC for the tetra- (OmcE, A3MW92, and F2KMU8), hexa- (OmcS), and octaheme (OmcZ) CNs. The figure also compares the energy matrices for the two CryoEM models of OmcS (PDB 6EF8 and 6NEF) and OmcZ (PDB 7LQ5 and 8D9M), as well as the results for both of these proteins to a prior quantum mechanics/molecular mechanics investigation that used MD-sampled configurations in different redox microstates but *assumed* no heme-heme interactions (Guberman-Pfeffer, 2023b).

The thermodynamic description of a multiheme protein, let along all structurally characterized CNs, is extremely demanding computationally with quantum mechanics (Barrozo et al., 2018) and experimentally requires advanced nuclear magnetic resonance techniques that have yet to be extended to a system with as many redox centers as the CNs (Silva et al., 2023). Figure 4 therefore showcases the insights currently only available via the systematic series of calculations implemented in BioDC using the PBSA method.

The diagonal site energies in Figure 4 are expressed relative to the most negative site, because only the *differences* between sites matter for the energetics of electron transfer. More positive values on the diagonal indicate an electrostatic destabilization of the oxidized versus reduced state and should correlate with higher (more positive) redox potentials.

The difference in relative site energies for adjacent hemes is predicted by BioDC to be ±0.26 eV for all filaments. The BioDC calculations on rigid structures yielded ranges of −0.16–0.19 eV for OmcS (PDB 6EF8) and −0.18 to 0.11 eV for OmcZ (PDB 7LQ5), closely matching the respective ranges of −0.18 to 0.18 eV and −0.26 to 0.09 eV previously found from dynamical simulations paired with QM/MM techniques (Figure 4, panels E vs D and H vs G). For several other multiheme cytochromes studied to date, $\Delta G^{\circ }$ has likewise been found to be ≤|0.3| eV (Jiang et al., 2017; Jiang et al., 2019a; Jiang et al., 2020). Adjacent site energy differences are kept relatively small in multiheme cytochromes with otherwise identical cofactors in van der Waals contact because residues that electrostatically shift the redox potential of one heme also shift the redox potential of the adjacent heme to a similar extent and in the same direction.

The effect of shared electrostatic microenvironments on driving forces is further illustrated by considering redox-Bohr interactions, which have been measured for various triheme (Correia et al., 2002; Morgado et al., 2010; Dantas et al., 2012; Silva et al., 2021), tetraheme (Turner et al., 1996; Louro et al., 1998; Louro et al., 2001; Fonseca et al., 2009; Paquete and Louro, 2010), and nonaheme (Reis et al., 2002) proteins. Even when redox-Bohr center interactions cause substantial individual heme potential shifts, the maximum observed change in the *difference* between adjacent site energies remains small (≤0.04 eV). For example, in cytochrome c_3_ from *Desuljovibrio vulgaris*, interactions with redox-Bohr centers near heme I shift the potentials of hemes I, II, III, and IV by −0.070, −0.030, −0.018, and −0.006 V respectively (Turner et al., 1996). The *differences* between adjacent hemes (|≤0.040|V) remain comparable to thermal fluctuations at 300 K. Such small differential effects on $\Delta G^{\circ }$ would produce, at most, a factor of 3 change in the electron transfer rate for typical values of $\sqrt{\langle H^{2}\rangle }$ and λ (0.005 and 0.5 eV, respectively), for the computed $\Delta G^{\circ }s$ in the ±0.3 eV range.

The shared electrostatic microenvironments for adjacent hemes due to dense packing minimizes the effect of redox-Bohr interactions on driving forces. The anticipated magnitude of the effect, based on experimental characterizations of other multiheme cytochromes, does not change the conclusions of this work.

#### 3.2.2 Adjacent heme-heme interactions are ≤0.12 eV

Close-packing, however, introduces heme-heme redox anti-cooperativities. Figure 4 shows that the off-diagonal terms for first-nearest neighbor hemes are predicted to be 0.05–0.14 eV for all CNs, whereas more distant interactions are comparable to thermal energy. Several lines of evidence support this finding.

(1) BioDC previously described quantitatively the role of heme-heme interactions in water-soluble as well as membrane-embedded *de novo* designed heme protein maquettes (Hardy et al., 2024), and reproduces with the same accuracy the experimental thermodynamic description (Morgado et al., 2010) of the triheme Periplasmic protein cytochrome A from *G. sulfurreducens* (Supplementary Figure 1).
(2) Prior constant pH and redox molecular dynamics simulations (Guberman-Pfeffer, 2022) found interaction strengths of ∼0.08 eV by noting the difference in potentials when hemes were titrated simultaneously versus independently with all other hemes held in the oxidized state.
(3) Excitonic couplings between hemes computed at the QM/MM level (Figure 2B) are almost entirely Coulombic and agree quantitatively with the range of redox anti-cooperativities computed with classical electrostatics. These two calculations model distinct physical processes where the fate of the electron is different, but both create a positive hole that electrostatically interacts with the neighboring heme. The similarity of this electrostatic interaction is why excitonic couplings and redox anti-cooperativities are of the same magnitude. Oxidation, in a sense, is the limiting extent of a photo-excitation.
(4) A long established Debye-Hückel shielded electrostatics (DHSE) model (Fonseca et al., 2012) predicts similar, albeit smaller, interaction energies of 0.03–0.06 eV for the Fe-to-Fe distances found in the CNs. However, this model assumes an effective dielectric constant of 8.6, whereas the dielectric constants of the heme binding sites in the CNs are estimated by Equation 3 to be 5.2–7.6 (Section 2.2); a previous study found dielectric constants of 3–7 for OmcS (Guberman-Pfeffer, 2022). A smaller dielectric constant means electrostatic interactions are less screened, and the interaction energies should therefore be larger, as found here.
(5) A recent spectroelectrochemical experiment on OmcS was *interpreted* to mean heme-heme interactions are negligible (Portela et al., 2024), but for hemes in van der Waals contact, this interpretation is physically unreasonable and relies on a fundamental misunderstanding of statistical mechanics.

As the solution potential is swept in a spectroelectrochemical experiment, a multi-center redox protein passes through a series of macroscopic oxidation stages (Paquete and Louro, 2014). Each stage comprises an ensemble of microstates that describe all possible ways of distributing reducing equivalents among the redox centers. The potentials at which these macroscopic transitions occur are observed by spectroelectrochemistry. But from those potentials, it is not possible to deduce anything about the statistical weights of the microstates that produced the bulk-averaged observable. The finding that a model of independent Nernstian centers fits the titration curve does not rule out the presence of heme-heme interactions any more than the equally good fit of a model with sequentially-coupled Nernstian centers confirms their presence.

Furthermore, the prior report (Portela et al., 2024) used a 6-parameter fit of independent or sequentially-coupled redox reactions to account for the titration curve without any sensitivity analysis to guard against overfitting. A re-analysis of the titration curve for OmcS (Supplementary Figure 8) indicates that a model of only 3 hemes is sufficient to fit the curve according to either the Akaike or Bayesian information criterion, which balances model fit against model complexity, or even the plateauing of the root-mean-squared-error of the fit with the increasing number of hemes in the model.

At the low resolution of the smooth experimental titration curve, half (3/6) of the hemes in a subunit of OmcS appear to have potentials indistinguishable from the potentials of the other hemes, and the full range of potentials is only ∼0.2 V. The small differences and overall range are in good agreement with the prediction of relative site energies in Figure 4.

Worth noting in passing because the original authors did not: The spectroelectrochemical data definitively invalidates their prior models of why OmcS is more conductive upon cooling or chemical reduction. Malvankar and co-workers predicted a massive hysteresis between cathodic and anodic directions (a 0.4 V shift in midpoint potential), as well as potentials as low as −0.826 V versus standard hydrogen electrode (SHE) (Dahl et al., 2022). Neither prediction, published in 2022, was born out (Portela et al., 2024), even by earlier spectroelectrochemical data collected in 2020 (O’Brien, 2020) and 2021 (Shipps, 2022) under the supervision of Malvankar.

#### 3.2.3 Linear filament topology blunts the effect of redox anti-cooperativity on driving forces

**FIGURE 5:**
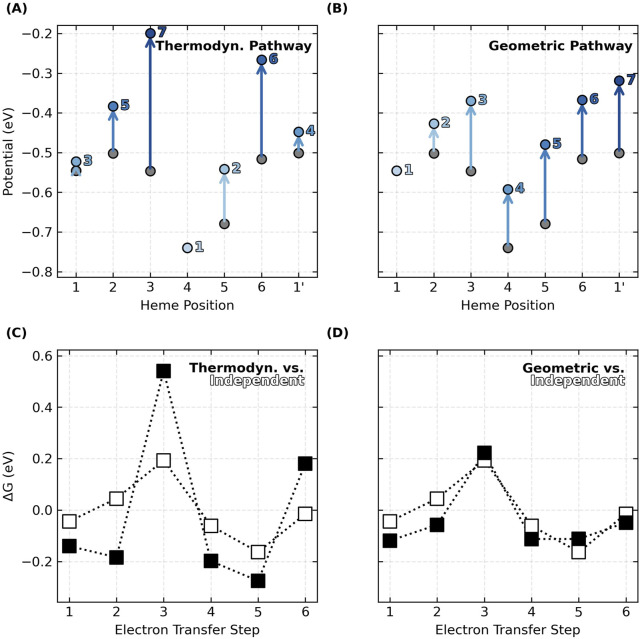
Oxidation of hemes in the linear sequence prescribed by the topology of cytochrome “nanowires”, as opposed to the thermodynamically preferred order, blunts the effect of redox anti-cooperativities on the $\Delta G^{\circ }$ landscape for electron transfer. **(A, B)** Total shifts in oxidation energies due to sums of anti-cooperative redox interactions as the hemes oxidize in order of their thermodynamic preferences or geometric arrangement. The x-axis follows the linear sequence of the filament whereas the light-to-dark blue annotations indicate the oxidation order. **(C, D)** Comparisons between the landscapes of anti-cooperatively interacting hemes (oxidizing in either thermodynamic or geometric order) and a reference landscape of hypothetically non-interacting hemes oxidizing independently. The data shown is for OmcS. Analogous figures for the other cytochrome “nanowires” are Supplementary Figures 9–15.

The relative oxidation energies on the diagonal of each energy matrix in Figure 4 do *not* monotonically increase in the geometric order of hemes #1 to N in the CNs. This observation means that the preferred thermodynamic oxidation sequence is *different* from the oxidation order that must happen as electrons flow linearly along the heme chain. If the oxidation of each heme shifts the potential of the first nearest neighbor by ∼+0.1 V because of anti-cooperative interactions, oxidizing the hemes in geometric versus thermodynamic order has a significant consequence (Figure 5).

The predicted thermodynamic order for heme oxidations in OmcS, for example, is #4, #5, #1, #1′, #2, #6, and #3 (Figure 5A), where the prime mark indicates the first heme of the next subunit. This ordering (except for the position of Heme #1) matches a prior QM/MM investigation (Guberman-Pfeffer, 2023b).

But the specific ordering is less important here than the fact that some hemes become oxidized *after* the flanking heme on either side has already been oxidized. The result is that heme-heme interactions compound, and the middle heme oxidizes at a much more positive potential. This is what happens for Heme #3 (oxidizing after Hemes #2 and #4) and Heme #6 (oxidizing after hemes #5 and #1′) in OmcS (Figure 5A). The consequence is that the peaks and valleys of the free energy landscape are accentuated relative to if the hemes oxidized independently with no heme-heme interactions (Figure 5C).

If, instead, hemes oxidize in order of geometric arrangement (Figure 5B), the potential of each and every heme shifts by a similar amount from the preceding oxidation of the heme immediately before it in the sequence. With every heme shifting by roughly the same amount as a cascade of oxidations moves through the linear topology of the CN, the potential differences, and free energy landscape are largely unaffected (Figure 5D).

The same conclusions are reached for all examined CNs (Supplementary Figures 9–15). Thus, by virtue of having a linear topology that constrains the hemes to oxidize in a geometric sequence different from thermodynamic preferences, potential shifts due to heme-heme interactions do not compound, and the free energy landscape for multi-step electron transfer is rendered largely robust to redox anti-cooperativities.

### 3.3 Reorganization energy

Electron transfer reorganization energy is generally dissected into inner- $\left(\lambda_{\text{in}}\right)$ and outer- $\left(\lambda_{\text{out}}\right)$ sphere components. $\lambda_{\text{in}}$ reflects oxidation-state dependent changes in internal coordinates that, for a heme group, contribute 0.05–0.08 eV to the activation barrier (Sigfridsson et al., 2001; Tipmanee et al., 2010). $\lambda_{\text{out}}$ reflects the energetic penalty for rearranging the environment to accommodate altered charge distributions on the electron donor and acceptor.

**FIGURE 6:**
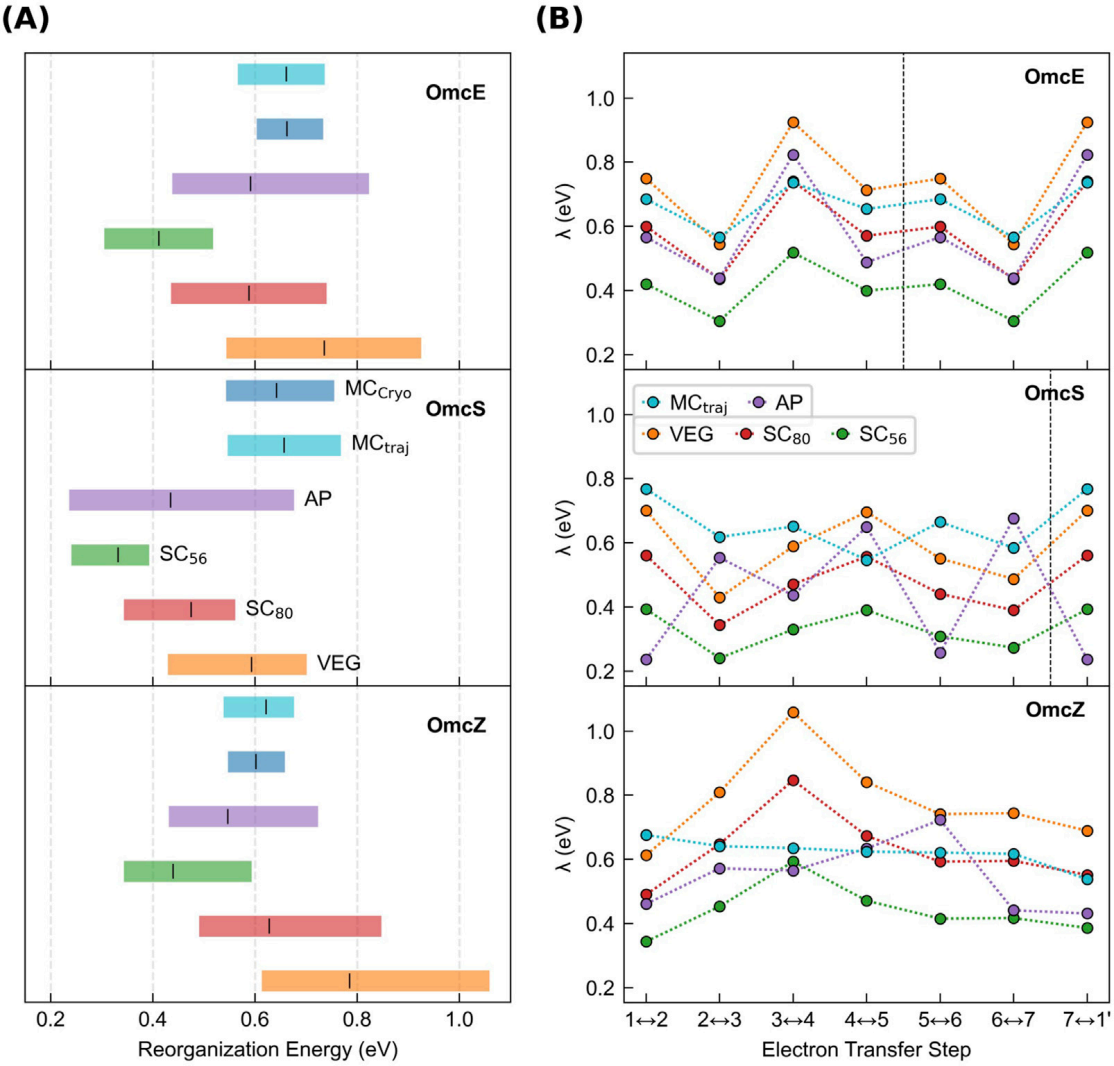
Outer-sphere reorganization energies in the cytochrome “nanowires” are always tenths of an eV regardless of whether or how electronic polarizability is considered. **(A)** Range of $\lambda_{\text{out}}$ for electron transfers through Omc- E, S, and Z according to different methods. **(B)** Variation in $\lambda_{\text{out}}$ as a function of the position along the chain and the various computational methods. **(A, B)** MC_Cryo/traj_, VEG, SC_80_, SC_56_, and AP respectively indicate $\lambda_{\text{out}}$ was estimated from an empirically parameterized form of the Marcus continuum equation applied to either the CryoEM geometries or molecular dynamics trajectories, Coulombic vertical energy gaps, those gaps scaled by factors of 0.80 or 0.56, or combined with the perturbatively-computed polarization energy for the active-site. Vertical dashed lines mark subunit boundaries to allow comparisons across filaments with fewer electron transfer steps per subunit than OmcZ. Data is repeated after the boundary because of the homopolymeric nature of the filaments.

To quantify $\lambda_{\text{out}}$ , various methods have been proposed that account for the influence of electronic polarizability in different ways and to different extents. Measurements on cytochrome *c* have served as a reference, but experimental values vary by as much as ∼0.5 V depending on conditions (Alvarez-Paggi et al., 2013; Chattopadhyay et al., 2021). Different approaches have either reached the same level of agreement (Dinpajooh et al., 2016; Seyedi et al., 2017; Jiang et al., 2019b; Martin et al., 2019; Futera et al., 2020) or been judged successful relative to different experimental values (Daidone et al., 2014; Martin et al., 2019). To clarify matters for CNs and hemoproteins more generally, different approaches for computing $\lambda_{\text{out}}$ are compared for three of the CNs (Section 3.3.1) before one of the methods is used to compare the full set of CNs (Section 3.3.2). The results are summarized in Figure 6 and Supplementary Tables 3–5.

The following discussion shows that all methods, with or without including redox-linked conformational dynamics, gave $\lambda_{\text{out}}$ between 0.24 and 1.06 eV. Since these values are ≥20-fold larger than $\sqrt{\langle H^{2}\rangle }$ (Section 3.1), standard Marcus theory is the appropriate theoretical framework for describing electron transfer. The prior proposal of coherence-assisted transport predicated on unusually and uniformly low (∼0.15 eV) reorganization energies in OmcS (Eshel et al., 2020) can be put aside.

#### 3.3.1 Coulombic vertical energy distribution approach

$\lambda_{\text{out}}$ was estimated from the distributions of vertical energy gaps (VEGs) in the reactant and product states for each heme-to-heme electron transfer in CNs previously submitted to MD simulations, namely, Omc- E, S, and Z. The distributions were reasonably Gaussian in every case (Supplementary Figures 2–4) and showed little sign of non-ergodic behavior: The ratio of the variance to Stokes shift reorganization energy was within ±0.2 of unity (Supplementary Table 3). This behavior was similar to what was previously found for cytochrome *c*, but differs from studies on plastocyanin, the bacterial reaction center, green fluorescent protein, the bacterial bc_1_ complex, and bacterial complex I (Martin et al., 2019). $\lambda_{\text{out}}$ , taken as $\lambda^{\text{rxn}}$ in Equation 7, was (Figure 6, “VEG”) 0.544–0.925 (OmcE), 0.429–0.701 (OmcS), and 0.613 (OmcZ) eV.

#### 3.3.2 Empirically corrected vertical energy gaps for medium polarizability

To account for electronic polarization of the environment in response to oxidation-state cycling at the active site, recommended scaling factors (SC) of 0.80 or 0.56 were applied to $\lambda_{\text{out}}$ from the VEG approach (Kontkanen et al., 2022; Matyushov, 2023). The resulting ranges for Omc- E, S and Z “nanowires” were 0.435–0.740, 0.343–0.561, and 0.490–0.847 (Figure 6; SC_80_), or 0.305–0.518, 0.240–0.393, and 0.343–0.593 (Figure 6; SC_56_) eV.

#### 3.3.3 Perturbatively corrected vertical energies for active site polarizability

An alternative approach to correct for the polarizability of the heme active site was used in which the VEGs are expanded by perturbation theory to second order as a sum of Coulombic and polarization energies (Martin et al., 2019; Futera et al., 2020). The polarization energies depend on the change of the polarizability tensor for the heme group between the oxidized and reduced states $\left(\Delta \alpha \right)$ and the electric field $\left(E\right)$ exerted on the heme by the protein-water environment.

##### 3.3.3.1 Heme polarizability

Δα was computed for His-His ligated hemes, both in vacuum and in the distinct protein-water binding sites of OmcS. Supplementary Tables 1 and 2 show that Δα was minimally changed from its vacuum value by the protein-water environment and thermalization during MD, as well as replacement of the distal His ligand with Met. The minor deviations from the *in vacuo* results due to static as well as dynamic conformational and environmental heterogeneity can be neglected for simplicity. The analysis therefore focuses on the *in vacuo* results for the His-His ligated *c*-type heme found in all known CNs and makes comparisons to the literature on the His-Met ligated heme of cytochrome *c* (Dinpajooh et al., 2016; Jiang et al., 2019b). The results are proposed to be general for both His-His and His-Met ligated *c*-type hemes in any hemoprotein.

**FIGURE 7:**
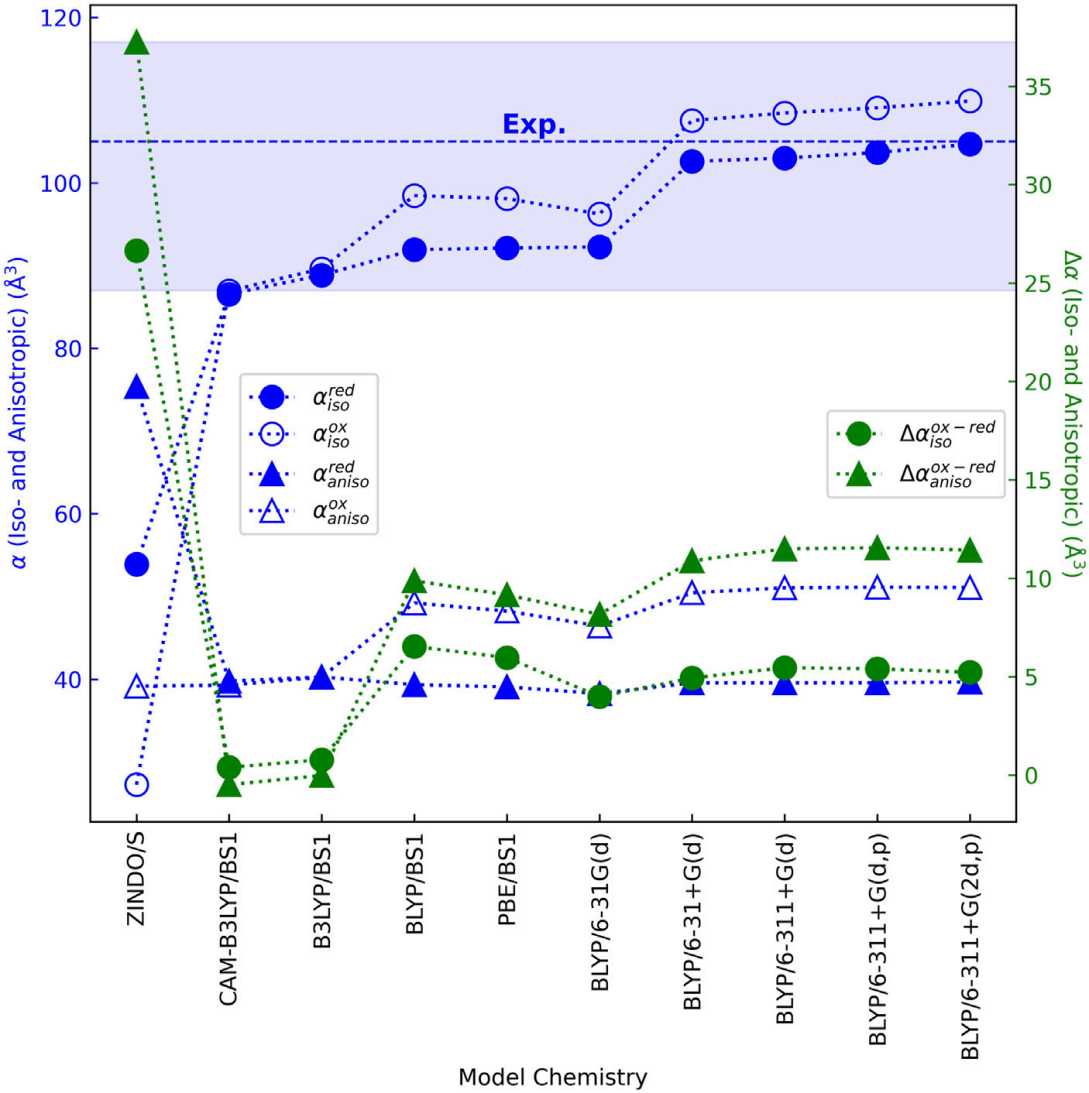
Validated and converged quantum chemistry models show that a bis-histidine ligated *c*-type heme is similarly polarizable in the reduced and oxidized states, far more so than previously predicted by the unvetted ZINDO/s method for this purpose (Martin et al., 2019; Futera et al., 2020). The average (dashed line) and standard deviation (shaded region) of the experimental isotropic polarizability for the heme memetic tetraphenylporphyrin-iron (III) chloride are shown (Deachapunya et al., 2007). Neither the isotropic polarizability in the reduced state nor the anisotropic polarizability in either oxidation state was reported.

DFT techniques have been found to accurately reproduce the (iso/aniso)-tropic polarizabilities of rigid to semi-rigid molecules (Hickey and Rowley, 2014; Hait and Head-Gordon, 2018; Pabst and Blochowicz, 2022), including a heme memetic (Deachapunya et al., 2007). Using these methods and ensuring basis set convergence on the results, Figure 7 (Supplementary Table 1) shows that the isotropic polarizability $\left(\alpha_{\text{iso}}\right)$ of the heme cofactor is 86–104 Å^3^ in either oxidation state regardless of the approximate density functional and basis set combination employed.

The range for $\alpha_{\text{iso}}$ fell within the experimental uncertainty for the heme memetic *meso*-tetraphenyl-iron (III) porphyrin (Deachapunya et al., 2007), and exceeded the prior estimates of 54 and 27 Å^3^ in the reduced and oxidized states, respectively, from the Zerner’s Intermediate Neglect of Differential Overlap with Singles (ZINDO/S) method applied to a His-Met ligated *c*-type heme (Dinpajooh et al., 2016; Jiang et al., 2019b).

The change in isotropic polarizability between oxidation states $\left(\Delta \alpha_{\text{iso}}\right)$ was found by DFT methods to be at most 6.5 Å^3^, which is also independent of environment and ligand set. The obtained $\Delta \alpha_{\text{iso}}$ was 4-fold smaller than that previously predicted by ZINDO/s, but it is in good agreement with the estimate obtained by analyzing the UV-vis spectra of cytochrome *c* in both redox states (Sarhangi and Matyushov, 2023). Spectral analysis from 600 to 180 nm found no significant absorption intensity outside the visible range that would change this estimate (Urry and Pettegrew, 1967).

A similar story applies to the anisotropic polarizability $\left(\alpha_{\text{aniso}}\right)$ . DFT methods predict $\alpha_{\text{aniso}}$ to be ∼40 and ∼50 Å^3^ in the reduced and oxidized states, respectively, giving $\Delta \alpha_{\text{aniso}}$ of ∼10 Å^3^. Prior ZINDO/s calculations on a His-Met ligated heme, by contrast, predicted 75 and 39 Å^3^ in the reduced and oxidized states, respectively, with a difference, again, four-fold larger than found by DFT methods.

Small changes in Δα are consistent with small geometric changes (*e.g.*, ∼0.01 Å and <1° in bond lengths and angles, respectively, at the BLYP/6-31G(d) level of theory) between the redox states and the low internal reorganization energy for the heme group. The discrepancy between ZINDO/s and DFT most likely reflects the inadequacy and unreliability of the former method for predicting polarizabilities. The use of ZINDO/s for this purpose requires summing contributions to the polarizability from vertical excitations computed up to 10 eV above the ground state (Dinpajooh et al., 2016). As the sum over excited states must grow to large (e.g., 100) numbers (Dinpajooh et al., 2016) to converge the polarizability, increasingly larger errors accumulate when using a semi-empirical method parameterized to reproduce only the lowest-lying excited states. Furthermore, ZINDO/s, unlike DFT, has not been parameterized or benchmarked against experiment for computing polarizabilities.

The overall conclusion is that: Oxidation-state dependent changes in iso- and anisotropic polarizabilities for the heme cofactor are predicted by DFT to be far *smaller* (<15 Å^3^) than previously found with ZINDO/s semi-empirical theory, regardless of different axial ligands, molecular environments, or chosen DFT model chemistries.

##### 3.3.3.2 Protein-water electric fields

The protein matrix for all structurally characterized CNs displays a formal net negative charge, where “formal” refers to the assumption of standard pK_a_s for titratable residues. On a per-subunit basis of the homopolymeric filaments, the formal net charges are −3*e* (A3MW92 and OmcS), −6*e* (OmcE), −8*e* (OmcZ), and −11*e* (F2KMU8).

Despite the almost quadrupling of per-subunit formal net charge, the heme-Fe centers in an interior subunit of all CNs experience a similar but wide range in electric field magnitudes from 0.07 to 0.7 V/Å (Supplementary Table 6). Across the binding sites in a given protein, the field magnitudes $\left(\left|E\right|\right)$ are 0.34 ± 0.13/0.37 ± 0.10 (OmcZ), 0.36 ± 0.20 (A3MW92), 0.42 ± 0.22/0.36 ± 0.22 (OmcS), 0.43 ± 0.15 (OmcE), and 0.52 ± 0.12 (F2KMU8), where the “/” indicates results for the two CryoEM structures of OmcS (PDB 6EF8 and 6NEF) and OmcZ (PDB 7LQ5 and 8D9M).

**FIGURE 8:**
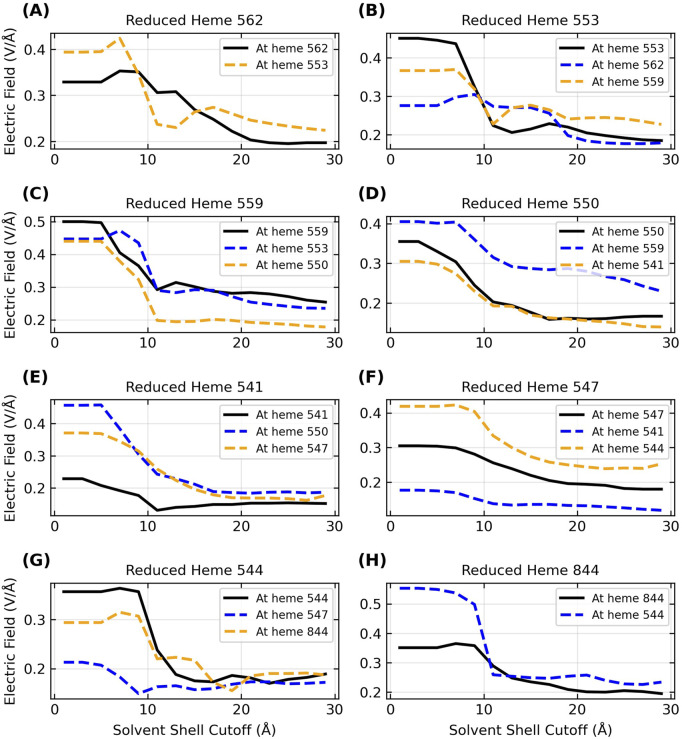
Inclusion of the explicit aqueous electrolyte with an increasing solvent cutoff causes a sizable dampening in the strength of the electric field exerted on the heme-Fe centers of OmcZ for the reduced donor (black curve) and the oxidized acceptor (blue/orange curve) heme on either side of it in the chain. The panels **(A–H)** in alphabetical order track the movement of an electron through the filament where the sequence of electron donors in terms of the residue IDs is 562 → 553 → 559 → 550 → 541 → 547 → 544 → 844.

These field magnitudes were calculated [as done previously for heme systems (Bím and Alexandrova, 2021)] directly on the CryoEM structures after geometry optimization, and therefore do not include conformational dynamics or solvation. For the three filaments simulated by MD in an explicit aqueous solvent with sufficient counterions for overall charge neutrality (Omc- E, S, and Z), the solvent typically suppressed $\left|E\right|$ exerted by the protein matrix. Figure 8 shows the dependence of $\left|E\right|$ in OmcZ on the cutoff distance for including the solvent; analogous figures for Omc- E and S are shown in Supplementary Figures 15, 16.

The range of $\left|E\right|$ shrank and shifted to lower values for each protein as more of the solvent was included. For the reduced state of each heme in OmcZ (black curves in Figure 8), for example, the range of $\left|E\right|$ changed from 0.23 to 0.50 V/Å at a 1.0 Å cutoff to 0.15–0.25 V/Å at a 29 Å cutoff for the solvent (Supplementary Table 7). The conclusion is that the hydration state of CNs significantly affects the magnitude of the electric field experienced at the heme-Fe centers.

Since the solvent dampens the electric field exerted by the protein, and the total field experienced by the electron influences the activation barrier for the redox reaction, it follows that physiological redox conduction through a protein is sensitive to the hydration state. This fact is well known (Phan et al., 2016), and a prior study proposed that changes in the solvent microenvironment around OmcS may contribute to its anti-Arrhenius conductivity behavior (Guberman-Pfeffer, 2022). The observation of anti-Arrhenius kinetics is inconsistent with thermally activated, biologically relevant redox chemistry in cytochromes (Malvankar et al., 2011; Malvankar and Lovley, 2012). A study that attempted to rationalize the kinetics with this mechanism (Dahl et al., 2022) has neither been supported by a more rigorous theoretical treatment (Guberman-Pfeffer, 2022) nor experimental evidence (Portela et al., 2024). The observation therefore remains unexplained and awaits a sound theoretical explaination.

In articulating the fact that hydration state matters for redox conductivity, it is clear that all electrical characterizations to date on air-dried CNs are biologically irrelevant, even apart from the known structural distortions that likely occur upon dehydration (Baquero et al., 2023). But why, then, are reported conductivities the same for hydrated and dehydrated samples of CNs (Yalcin et al., 2020)?

The simplest hypothesis is that electrons are not conducted by a biological redox cascade in either state because of the chosen experimental technique. That is, the residence time of the electrons may be so short that there is insufficient time for the solvent to screen the changing electrostatic potential along the protein. Whether the solvent is absent or frozen on the timescale of electron transfer, it cannot participate in the process. If the solvent does not participate, the process is abiological electron transport, not biological electron transfer (Bostick et al., 2018).

In support of this conclusion, the same electrical measurements currently attributed to filamentous cytochromes (Wang et al., 2019; Yalcin et al., 2020) were argued to be incompatible with cytochromes before the CryoEM structures were solved (Adhikari et al., 2016; Tan et al., 2016a). Given that CryoEM has shown the filaments to be cytochromes (Wang et al., 2019; Wang et al., 2022a; Gu et al., 2023), spectroelectrochemistry has confirmed their redox activity (Portela et al., 2024), and ground-state biological electron transfer is well-known to be redox-mediated, electrical measurements that have always been so inconsistent with redox chemistry that they required invoking a “new paradigm” of metallic-like conductivity (Malvankar and Lovley, 2012; Malvankar et al., 2012) have simply never been biologically relevant.

**FIGURE 9:**
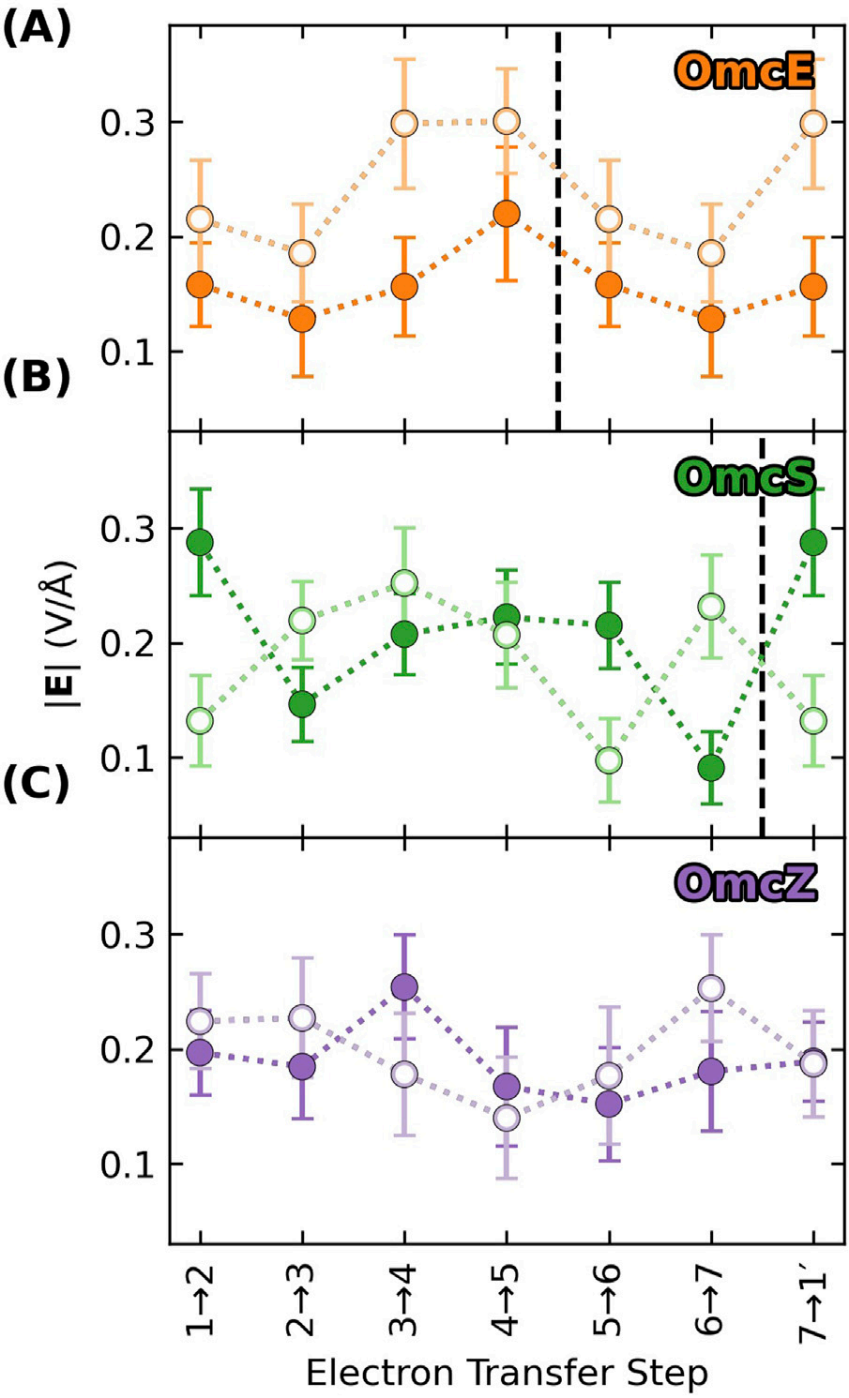
The electric field exerted on the donor (filled) and acceptor (unfilled) for a given electron transfer step is not identical, which means that the oppositely signed polarization energies for the two redox partners will not perfectly cancel, and the net result will contribute to the reorganization energy. Each panel **(A–C)** shows data for a different cytochrome “nanowire.” Vertical dashed lines mark subunit boundaries to allow comparisons across filaments with fewer electron transfer steps per subunit than OmcZ. Data is repeated after the boundary because of the homopolymeric nature of the filaments.

Returning to the discussion of electric fields in the context of electronic polarizability and reorganization energy, Figure 9 shows a key result: The electric field in any of the CNs is similar but not identical in magnitude (as well as direction) at the heme-Fe centers of the donor (filled circles) and acceptor (unfilled circles) at each electron transfer step. Differences in the field along the heme chain can couple to the equal and oppositely signed Δα for oxidation of the donor and reduction of the acceptor to produce net polarization energy and thereby change $\lambda_{\text{out}}$ .

##### 3.3.3.3 Active site polarized $\lambda_{\text{out}}$

**FIGURE 10:**
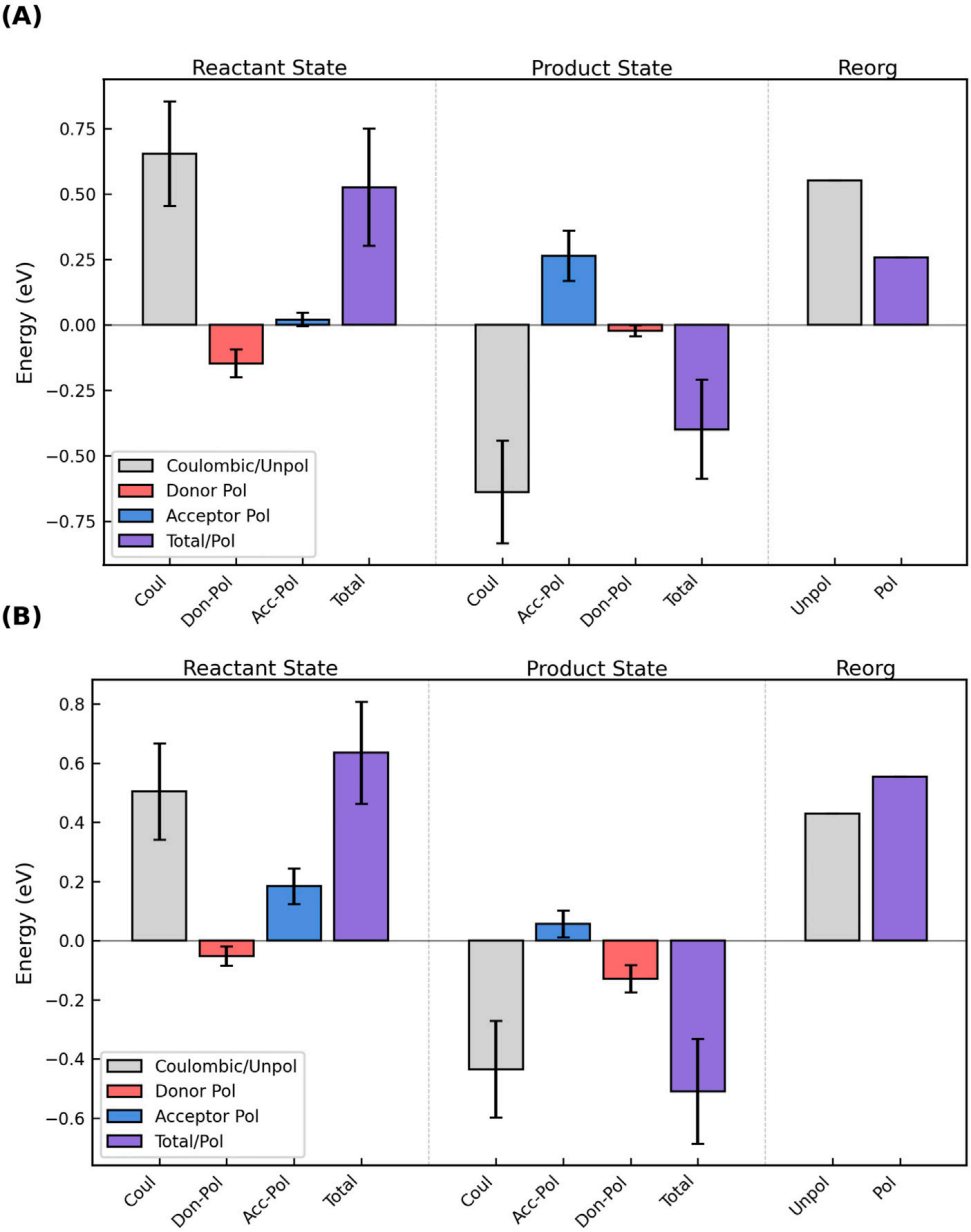
The oppositely signed polarization energies for oxidation of the donor and reduction of the acceptor in the reactant and product states can **(A)** decrease or **(B)** increase the total vertical energy gap and respectively cause a decrease/increase in the outer-sphere reorganization energy. The figure shows the Coulombic energy (gray), the separate polarization energies of the donor (red) and acceptor (blue), and the sum of those three terms (purple) for the reactant and product states, as well as the unpolarized (gray) and polarized (purple) reorganization energies for the electron transfer. The figure shows the results for **(A)** Heme #5 → #6 and **(B)** Heme #2 → #3 in OmcS; analogous figures for all electron transfer steps in Omc- E, S, and Z are shown as Supplementary Figures 18–34.

Generally speaking, the polarization energy computed by Equation 10 for Omc- E, S, and Z was negative (stabilizing) for the donor and positive (destabilizing) for the acceptor. Figure 10A shows the most common case: The donor was stabilized more than the acceptor was destabilized in the reactant state, and the effect was to decrease the magnitude of the total (Coulombic + donor polarization + acceptor polarization) VEG for the forward electron transfer. Conversely, the acceptor was destabilized more than the donor was stabilized in the product state, which decreased the magnitude of the total VEG for the reverse reaction. The result was a lowering of $\lambda_{\text{out}}$ . This active-site-polarization effect (shown in Figure 6 as “AP”) lowered $\lambda_{\text{out}}$ by 0.11–0.23 (OmcE), 0.05–0.47 (OmcS), and 0.02–0.49 (OmcZ) eV (Supplementary Table 3).

For two electron transfer steps, both in OmcS (Heme 2→3 and 6→1′, where the prime indicates the heme from the next subunit), the destabilization of the acceptor in the reactant state and the stabilization of the donor in the product state exceeded, respectively, the stabilization of the donor in the reactant state and the destabilization of the acceptor in the product state (Figure 10B). The result was an increase in the VEGs for the forward and reverse reactions, and a larger $\lambda_{\text{out}}$ .

Figures analogous to Figure 10 are shown for each electron transfer step in Omc- E, S, and Z as Supplementary Figures 18–34.

$\lambda_{\text{out}}$ by this AP approach was 0.438–0.823 (OmcE), 0.236–0.676 (OmcS), and 0.431–0.723 (OmcZ) eV. Of note, the ergodicity factor changed with the inclusion of electronic polarizability from 0.9 to 1.2 for Omc- E, S, and Z to 1.3–1.5 (OmcE), 1.0–2.3 OmcS, and 1.2–1.7 (OmcZ) (Supplementary Table 3). The increased deviation from unity indicates a modest non-ergodicity that has been seen before, for example, for cytochrome *c* when polarizability is considered (Dinpajooh et al., 2016).

#### 3.3.4 Empirically parameterized Marcus Continuum approach

A much simpler approach to estimate $\lambda_{\text{out}}$ is to use a form of the Marcus continuum expression (“MC” approach) in which the static dielectric constant $\left(\epsilon_{s}\right)$ was previously parameterized as a linear function of the total (donor + acceptor) SASA (Jiang et al., 2019a). The approach is motivated by the facts that the solvent usually constitutes ∼50% of $\lambda_{\text{out}}$ (Tipmanee et al., 2010), and for hemoproteins, $\lambda_{\text{out}}$ is controlled by SASA (Bortolotti et al., 2011).

This approach (Equations 3 and 4 in *Methods*) only depends on structural parameters, which were measured from the CryoEM structures of the known CNs (Supplementary Figure 35A; Supplementary Table 4), as well as hundreds of nanoseconds of previously published MD simulations for three of these structures (Supplementary Figure 35B; Supplementary Table 5).

All adjacent heme pairs cluster around Fe-to-Fe distances of ∼9 and ∼11 Å, which are respectively characteristic of slip- and T-stacked packing geometries. The donor + acceptor SASA of adjacent heme macrocycles (not including propionic acid substituents) was typically <78 Å^2^. $\epsilon_{s}$ of the heme binding sites ranged from 5.2 to 7.6, or in more chemically intuitive terms, from being slightly more polar than chloroform $\left(\epsilon_{s}=4.8\text{ at }25^{{}^{\circ}}C\right)$ to as polar as tetrahydrofuran $\left(\epsilon_{s}=7.6\text{ at }25^{{}^{\circ}}C\right)$ solution.

The similar Fe-to-Fe spacings and macrocycle SASAs constrained $\lambda_{\text{out}}$ in all CNs to the narrow range 0.543–0.770 eV (Figure 6, “MC”). Thermal averaging caused relatively minor changes in Fe-to-Fe distances, donor + acceptor SASA, and $\lambda_{\text{out}}$ .

#### 3.3.5 Summary of the effect of polarizability on $\lambda_{\text{out}}$

$\lambda_{\text{out}}$ decreased by 0.105–0.225 eV (11%–31%) for OmcE using the AP versus VEG method. For OmcZ, $\lambda_{\text{out}}$ was reduced by 0.018–0.494 eV (2%–46%). OmcS gave a more complicated picture: $\lambda_{\text{out}}$ decreased by 0.047–0.463 eV (7%–67%), except for two electron transfers where it increased 0.125–0.189 eV (29%–39%). The conclusion is that the recommended scaling factors of 0.56–0.80 are generally in the appropriate range, but their universality does not hold.

For its simplicity and sufficient accuracy, the MC approach was mostly used for the comparative study of the structurally characterized CNs in the rest of this work. The uncertainty in activation energies and rates introduced by this choice is discussed below.

### 3.4 Intra-filament electron transfer activation energy

The activation energy $\left(E_{a}\right)$ in standard Marcus theory is defined as (Equation 12):

$$E_{a}=\frac{{\left(\Delta G^{\circ }+\lambda^{\text{rxn}}\right)}^{2}}{4\lambda^{\text{rxn}}}$$ (12)

**FIGURE 11:**
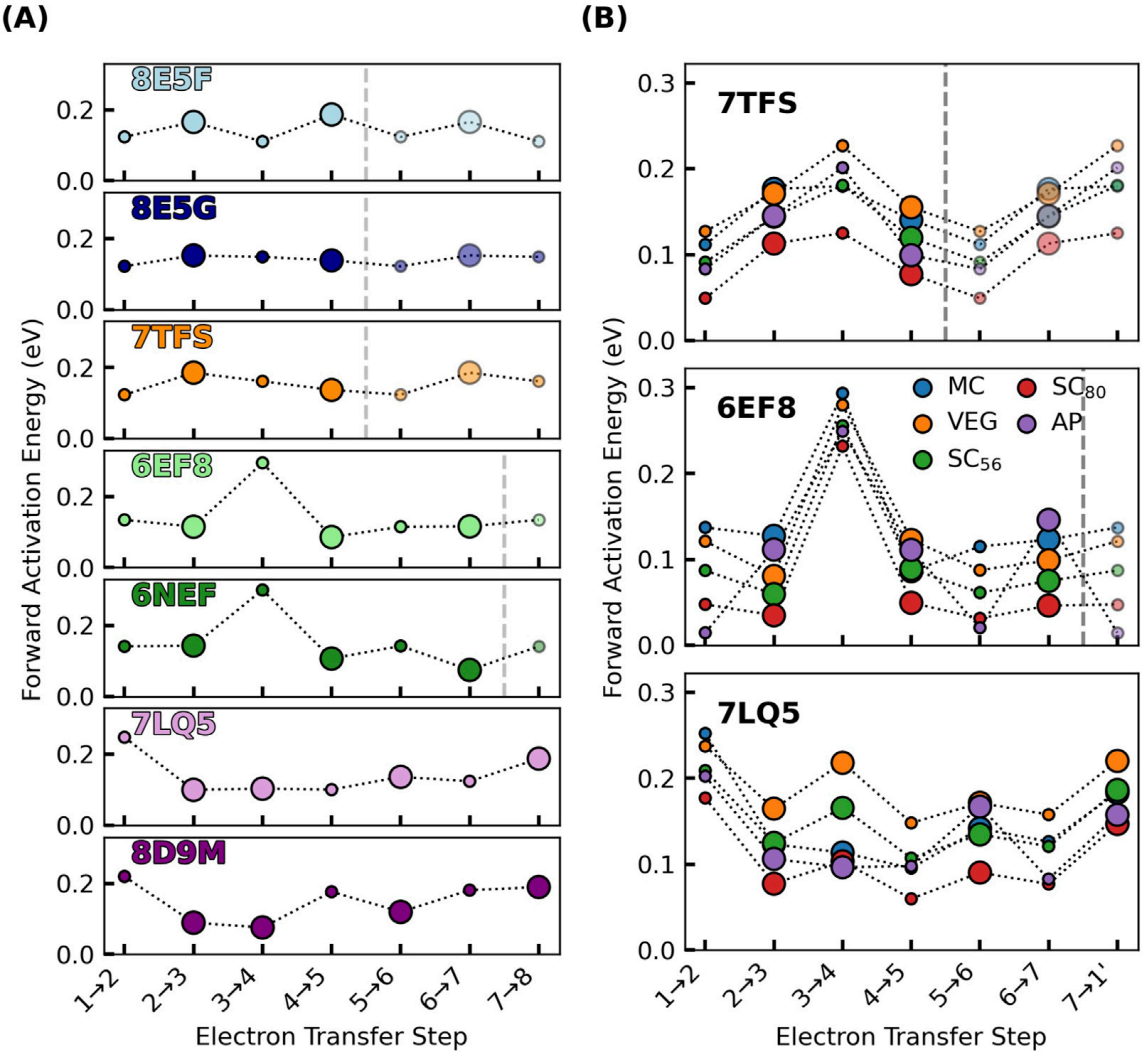
Activation energies for electron transfers in the known cytochrome “nanowires” are always ≤0.3 eV, regardless of if or how electronic polarization is included in the estimate of outer-sphere reorganization energy. **(A)** shows the cytochrome “nanowires” by PDB accession code: 8E5F, 8E5G, 7TFS, 6EF8/6NEF, 7LQ5/8D9M = A3MW92, F2KMU8, OmcE, OmcS, and OmcZ. **(B)** shows the variation in activation energy for three of these filaments for which the outer-sphere reorganization energy was computed by various methods discussed in the main text. The size of the markers in both panels is proportional to the electronic coupling. Activation energies for the forward reactions are shown; Supplementary Figure 36 shows the activation energies for the backward reactions. All data is presented in Supplementary Tables 8, 9.

Using $\Delta G^{\circ }$ s for the geometric oxidation sequence imposed by the linear topology of the CNs, $E_{a}$ is *always* ≤0.3 eV (Figure 11A; Supplementary Table 8). This result is insensitive to the method used to compute $\lambda_{\text{out}}$ (Figure 11B; Supplementary Table 9). Relative to the MC approach for $\lambda_{\text{out}}$ , the absolute average deviation in $E_{a}$ caused by the other methods are 0.027 (SC_80_), 0.033 (VEG), 0.037 (AP) and 0.058 (SC_56_) eV. Differences in $\lambda_{\text{out}}$ of this magnitude would change the computed rates in the next section by a factor of 10, but the conclusions would be unaffected.

With the MC approach for $\lambda_{\text{out}}$ , $E_{a}$ is 0.110–0.255 for A3MW92, F2KMU8, and OmcE, 0.075–0.300 eV for OmcS, and 0.076–0.248 eV for OmcZ. Thus, it appears that the range for $E_{a}$ is conserved among CNs.

### 3.5 Intra-filament electron transfer rates

**FIGURE 12:**
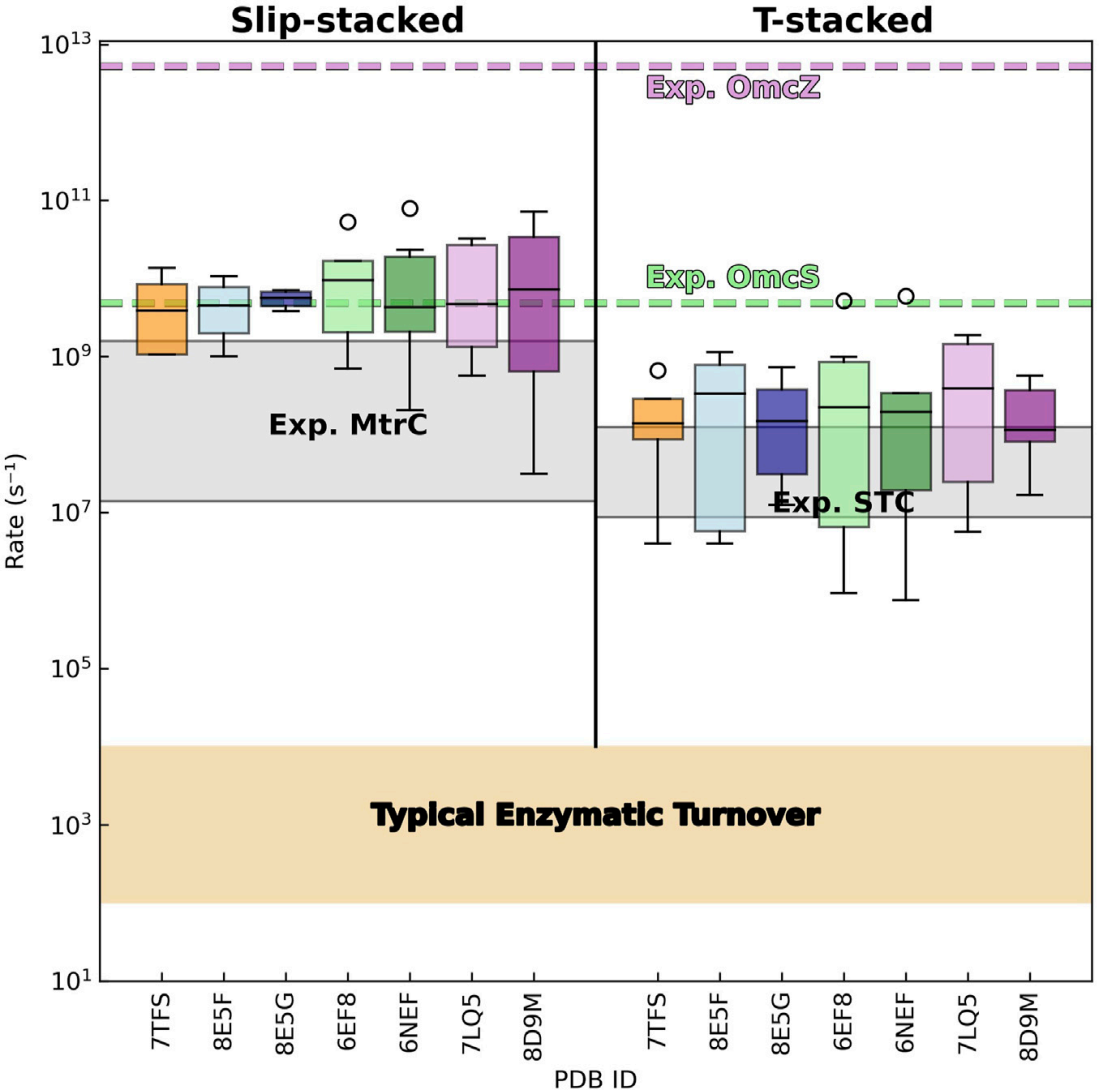
Electron transfer rates in cytochrome “nanowires” are not metabolically limiting, comparable to rates in other multihemes, and orders of magnitude slower than implied by the reported conductivities. Distributions of predicted (forward and backward) rates between slip- and T-stacked heme pairs are shown for the known cytochrome “nanowires.” The gray shaded regions show experimental rates reported for photosensitized variants of the metal reducing cytochrome (MtrC) (van Wonderen et al., 2021) and small tetraheme cytochrome (STC) (van Wonderen et al., 2019) from *Shewanella oneidensis*. The beige-shaded region covers the range of typical rates for enzymatic turnover (Noy et al., 2006). The dashed green and purple lines indicate the electron transfer rate that would be needed for every intra-wire heme-to-heme electron transfer to reproduce the previously reported conductivities of single Omc- S and Z filaments; see the main text for the derivation of these effective hopping rates.

Given the energetic parameters discussed in the preceding sections, the computed electron transfer rates within slip- and T-stacked heme pairs across all CNs cover (as expected) (Dahl et al., 2022) large and overlapping ranges (Figure 12; Supplementary Table 8): $3.1\times 10^{7}$ – $7.9\times 10^{10}$ s^-1^, and $7.5\times 10^{5}$ – $5.9\times 10^{9}$ s^-1^, respectively. However, the corresponding median rates across CNs reside in much narrower ranges of $3.8\times 10^{9}$ – $9.5\times 10^{9}$ and $3.4\times 10^{8}$ – $3.9\times 10^{8}$ s^-1^. This result suggests: (1) Typical rates within T-stacked pairs are at least an order of magnitude slower than within slip-stacked pairs; (2) The rates for specific heme packing geometries are roughly transferrable between proteins; and (3) The various CNs support similar electron fluxes (see Section 3.6). The following subsections put the computed rates into context with experimental expectations (Section 3.5.1) and the reported conductivities of the CNs (Section 3.5.2).

#### 3.5.1 Comparison to experimental expectations

The minimum, maximum, and median electron transfer rates predicted for the T-stacked geometry in all CNs are typically within a factor of ∼10 of the corresponding experimental rates for the same heme packing geometry in the small tetraheme cytochrome (STC) (van Wonderen et al., 2019). Predicted minimum, maximum, and median rates for slip-stacked hemes are typically within a factor of 60 (but in one case as high as 272) compared to rates measured in the metal reducing cytochrome (MtrC) (van Wonderen et al., 2021). As observed for the T- versus slip-stacked heme pairs in STC and MtrC, electron transfer rates in the former heme packing geometry were typically ∼10-fold smaller in the CNs.

The one-to-two order of magnitude agreement with experimental expectations should be weighed with the following considerations:

(1) The actual electron transfer rates in CNs are unknown. The comparison *assumes* that the rates are transferrable from different proteins for a given heme packing geometry. The similarity in the distributions of computed rates for different CNs in Figure 12 makes this assumption seem reasonable.
(2) The overestimation of slip-stacked rates relative to experimental expectations is inconsequential in the context of multi-step electron transfer: The slowest step in a multi-step reaction is rate-limiting, and ∼50% of the heme pairs in CNs are of the rate-throttling T-stacked variety. For this packing geometry, the predicted rates are in much closer agreement with experimental expectations.
(3) An order-of-magnitude uncertainty is introduced by the different methods for computing $\lambda_{\text{out}}$ . Additional uncertainty is introduced by neglecting the effect of redox-Bohr interactions on both $\Delta G^{\circ }$ and $\lambda_{\text{out}}$ . While redox-linked conformations are sampled for $\sqrt{\langle H^{2}\rangle }$ and $\lambda_{\text{out}}$ , they are only implicitly included when computing $\Delta G^{\circ }.$

(4) Even when using state-of-the-art MD and DFT methods to analyze electron transfer within the same protein, a 14-fold discrepancy between theoretical and experimental rates was reported (van Wonderen et al., 2019). The present study achieves comparable agreement despite using a more approximate method (BioDC) and comparing rates across different proteins, demonstrating the utility of this expedient approach for comprehensive analysis of all structurally-characterized CNs.

Inspired by the work of Blumberger and co-workers (Jiang et al., 2020), the BioDC program (Guberman-Pfeffer, 2023a) respectively estimates the reaction free energy, reorganization free energy, and RMS coupling from classical (PBSA) electrostatic calculations (Section 3.2), the empirically parameterized form of the Marcus continuum equation (Section 3.3.4), and the tilt angle between adjacent hemes (Section 3.1). These methods were applied only to the CryoEM geometries, although the independently solved structures for both Omc- S and Z gave similar results throughout this work.

Given only one or two geometries for each protein, the approximate methods used to compute the energetics of electron transfer, the exponential dependence of the rate on these energetics, the accepted order-of-magnitude error using a much more sophisticated protocol, and the fact that the actual rates in CNs are unknown, the obtained level of agreement with experimental expectations seems reasonable. It is also sufficient for the conclusions of this work.

#### 3.5.2 Comparison to reported electrical conductivities

As already noted, electron transfer rates in CNs have not been measured yet. However, an *effective* rate can be derived from the reported conductances $\left(G\right)$ (Yalcin et al., 2020; Dahl et al., 2022) via the Einstein-Smoluchowski relationship and standard electrical definitions (Equation 13).

$$k_{\text{hop}}=\frac{k_{b}T}{e^{2}}\frac{L_{w}}{Ar_{\text{DA}}^{2}\rho }G$$ (13)

The first term collects the Boltzmann constant $\left(k_{b}\right)$ , temperature $\left(T\right)$ , and elementary charge $\left(e\right)$ . The second term comprises physical properties of the CN; namely, the length $\left(L_{w}\right)$ and cross-sectional area $\left(A\right)$ of the conductor, the average charge-hopping distance $\left(r_{\text{DA}}\right)$ , and the charge density of the filament $\left(\rho \right)$ .

$L_{w}$ is taken as $3\times 10^{-5}$ cm because this is a typical electrode spacing used in characterization studies (Dahl et al., 2022). The heme chain at the core of each CN is approximately cylindrical, and the radius of the heme group is $7.5\times 10^{-8}$ cm (Eshel et al., 2020); A is therefore $\pi r^{2}=1.8\times 10^{-14}$ cm. The minimum edge-to-edge separation is the appropriate distance metric for heme-to-heme electron transfer (Moser et al., 2008), and for each CN $r_{\text{DA}}\approx 5$ Å. More specifically, for the only two CNs (Omc- S and Z) electrically characterized so far (Yalcin et al., 2020; Dahl et al., 2022), $r_{\text{DA}}$ is 4.86 (PDB 6EF8) – 5.01 (PDB 6NEF) $\times 10^{-8}$ cm for OmcS, and 4.40 (PDB 7LQ5) – 4.59 (PDB 8D9M) $\times 10^{-8}$ cm for OmcZ. The shortest average distance for each protein is used below.

ρ is given by the number of reducing equivalence per unit volume. Each heme can only be occupied by one reducing equivalent at a time, and the maximal conductivity is realized when 50% of the hemes are reduced, because in that state, there is an equal proportion of charge donors and acceptors. In the fully reduced (oxidized) state, there would only be electron donors (acceptors), and no electron transfer would be possible. This physical picture is one reason why the reported observation of 6-fold higher conductivity in the fully reduced versus fully oxidized state of OmcS (Dahl et al., 2022) does not make physical sense for biological, thermally activated, multi-step redox conduction, and thereby suggests these electrical characterizations are biologically irrelevant.

If ρ is taken for maximal conductivity, there are three or four reducing equivalence in a subunit of Omc- S and Z, respectively. The length of a subunit in these homopolymers is $4.7\times 10^{-7}$ and $5.8\times 10^{-7}$ cm, respectively, and A is the same because both have a conductive heme chain. Thus, ρ is 3.6–3.9 × $10^{20}$ charges/cm^3^.

A similar charge density was reported for filaments from *G. sulfurreducens* when they were argued to be composed of pili instead of cytochromes (Malvankar et al., 2014). It has been argued that this data should be re-interpreted *as if* it was measured on the now-known cytochrome OmcS (Wang et al., 2019), but the comparison is still flawed: The charge density was measured by subjecting the protein to a 10-V bias, and thereby accessed electronic states completely forbidden to biology.

With the discussed physical parameters, the reported conductances of $1.1\times 10^{-10}$ and $4.9\times 10^{-8}$ S (Siemens) for Omc- S and Z respectively give effective hopping rates by Equation 13 of $3.3\times 10^{10}$ and $1.4\times 10^{13}$ s^-1^. These rates are physically unreasonable for the multiheme architecture of CNs as described in the following paragraphs.

The effective $k_{\text{hop}}$ computed by Equation 13 assumes that uncorrelated electron transfers occur between sites of localization along a uniform chain. Operation of linear electron transfer chains, like those found in CNs are well described without site correlations, even in the presence of site-site interactions (Terai et al., 2023). But CNs are not uniform since every-other step is ∼10-fold slower than the preceding step. The effective rate for diffusion over alternatingly slow (T-stacked) and fast (slip-stacked) heme-to-heme rates cannot exceed the slowest rate. And yet, the $k_{\text{hop}}$ s implied by the experimental conductivities are 10^2^ and 10^5^-fold larger than the fastest known rate $\left(1.25\times 10^{8}{\;s}^{-1}\right)$ for the rate-limiting T-stacked packing geometry (van Wonderen et al., 2019).

The effective $k_{\text{hop}}$ for OmcZ is even more implausible since it is at the coupling-maximized ‘speed limit’ of 10^13^ s^-1^ proposed by Gray and Winkler (Gray and Winkler, 2010) for a van der Waals metal-to-metal contact distance of 3 Å. Electronic couplings in OmcZ are weak (Section 3.1) and the metal-to-metal distance is 9–11 Å (Wang et al., 2022a; Gu et al., 2023).

Given that typical enzymatic turnover (Noy et al., 2006) and interfacial protein-mineral electron transfer (Kerisit et al., 2007) are likely millisecond processes, there is no biological reason to make a filament with a $k_{\text{hop}}$ 10^7^, let alone 10^10^-fold faster.

These results complement the conclusion in Section 3.3.3.2 that the electrical measurements are biologically irrelevant. Some of the excessive rates may be due to the abiological nature of the electrical contacts with the protein (Polizzi et al., 2012; Baquero et al., 2023).

Unlike molecular redox partners in biology, the electrodes are not of molecular dimensions, and the observed current scales with the number of protein-electrode contacts. An intracellular protein can only donate a single electron at a time to a specific heme in the filament, but the atomic force microscopy tip used in characterization studies (Yalcin et al., 2020; Dahl et al., 2022) spans ∼10 subunits of the filaments and may inject electrons through the protein ‘insulation’ into as many as 80 hemes. The multiplicity of protein-nanoelectrode contacts in other measurements is unclear, but almost certainly greater than one (Baquero et al., 2023). The reported conductivities have not been properly scaled and normalized to account for multiple conductive channels for injecting electrons (Polizzi et al., 2012).

In nature, the filaments discharge electrons to an abiological mineral. There is no evidence that this interaction invalidates the CryoEM-resolved structures. However, deviations from the CryoEM structures have been attributed to experimental conditions such as drop-casting, air-drying, and crushing conductive CNs under an AFM tip on a denaturing bare gold electrode, particularly when 10-times more force is applied to the more flexible filament (Baquero et al., 2023; Guberman-Pfeffer, 2024). Additionally, abiological minerals in nature do not have the electronic band structure of laboratory metallic electrodes (Ahart et al., 2020).

A key insight of the present work is to put aside these non-biological measurements to instead ask if the computed conductivities of CNs suffice for their proposed role in cellular respiration.

### 3.6 Intra-filament electron flux

CNs are proposed to discharge the metabolic flux of electrons from a microorganism. Logical questions are then: What is the metabolic flux and do the conductivities of the CNs permit a bioenergetically reasonable number of them to serve this function? An upper bound for the single-filament conductivity needed for cellular respiration is derived in Sections 3.6.1–3.6.2, and the computed conductivities are compared to it in Sections 3.6.3–3.6.4. The metabolic discussion focuses on *G. sulfurreducens* since this model organism produces 3/5 of the structurally characterized CNs.

#### 3.6.1 Metabolic requirements versus solid state conductivities

A *G. sulfurreducens* cell respires at a rate of ∼10^6^ e^−^/s regardless of whether the terminal electron acceptor is an electrode (Jiang et al., 2013; Scarabotti et al., 2021), a metal salt (Chabert et al., 2020; Karamash et al., 2022), or a mineral (Lampa-Pastirk et al., 2016). Independent of the identity of the cation to be reduced (Ag^+^, Fe^3+^, Co^3+^, V^5+^, Cr^6+^, and Mn^7+^), the different redox potentials of the metal salts (∼0.21 V for AgCl versus ∼0.8 V for AgNO_3_ on the normal hydrogen electrode scale) (Chabert et al., 2020), or the downregulation/deletion of some cytochromes in the microbe, the rate of Fe^2+^-heme oxidation by the metal salts always equaled the rate of Fe^3+^-heme reduction by the respiratory machinery; what changed were the proportions of Fe^2+/3+^-hemes in the microbe. This result suggested that the electron efflux reflected the intrinsic respiratory rate that the cell must maintain for ATP synthesis. A similar rate of ∼10^5^ e^−^/s/cell was found for the bacterium *Shewanella oneidensis* (McLean et al., 2010; Gross and El-Naggar, 2015).

Note that the respiratory rate is independent of whether CNs were specifically expressed in the experiments. How electrons exit the cell is a different question from how many electrons need to exit the cell.

It is not true—in fact, just the opposite—that cellular respiration is more efficient if/when CNs are involved. When a microorganism must switch from using an intra- to extracellular electron acceptor, protons from the oxidation of organic matter are left behind to accumulate in the cytoplasm and reduce the proton motive force for ATP synthesis (Bird et al., 2011; Guberman-Pfeffer and Malvankar, 2021). Respiration, in fact, would entirely shutdown if it were not for mechanisms that have been found to remove at least some of the protons (Mahadevan et al., 2006; Firer-Sherwood et al., 2008; Silva et al., 2021). Also, no energy is realized for the microbe through EET (Bird et al., 2011): The electrons are de-energized ‘spent-fuel’ at the end of the electron transport chain in the inner membrane that simply needs to be discarded to make way for respiration to continue. Whether or not CNs help to discharge respiratory electrons when intra-cellular acceptors are not available, they do *not* power life.

*G. sulfurreducens* realizes the metabolic flux of ∼10^6^ e^−^/s at a low potential difference between the intra- and extracellular redox half-reactions. Voltametric experiments indicated that *G. sulfurreducens* reaches its maximum respiratory rate on anodes posed at −0.1 V versus SHE and does not take advantage of the additional potential energy available at higher potentials (Bond et al., 2012).

At an applied bias of −0.1 V vs SHE, an electron flux of $6.2\times 10^{6}$ e^−^/s, or 1.0 pA gives, by Ohm’s law a conductance of $9.9\times 10^{-12}$ S. This value is the total conductance the ‘circuits’ must have that connect the cell to extracellular electron acceptors.

If a *single* filament with the dimensions and charge density of Omc- S or Z (Section 3.5.2) were to discharge the *entire* metabolic current, its conductivity would need to be $1.7\times 10^{-2}$ S/cm. This is certainly an overestimate because (1) most studies indicate *G. sulfurreducens* generates ∼10-fold less current than the assumed 1.0 pA (Jiang et al., 2013; Chabert et al., 2020; Scarabotti et al., 2021; Karamash et al., 2022), and (2) *G. sulfurreducens* has alternate routes (e.g., porin-cytochrome complexes) for expelling electrons (Gralnick and Bond, 2023). Only a fraction of the metabolic current would need to be discharged by a CN. And yet, as a single-filament conductivity, it is 10^2^–10^5^-fold *smaller* than the conductivities reported for single Omc- S and Z filaments. Why would a bacterium ever need such highly conductive filaments?

A natural thought may be for communal growth. However, biofilms on electrodes serving as infinite electron sinks are likely laboratory artifacts with no relevance to the physiology of *G. sulfurreducens* in its normal habitat. If a natural electron sink, Fe(III) oxyhydroxide, occupies 50% of the space around a *G. sulfurreducens* cell, bioenergetic calculations (Levar et al., 2012) showed that the cell would need to reduce all available Fe(III) within a radius of 2–4 μm just to *approach* generating enough ATP for single-cell doubling. There is simply not enough oxidized mineral in a geographical area for *G. sulfurreducens* to support a multi-layer, microns-thick biofilm.

Living as a solitary microbe on a grain of sand (Levar et al., 2012), a *G. sulfurreducens* cell would have no use for the conductivities reported for its filaments. There are physical limits to the rate of intracellular acetate oxidation. For a single cell to make use of the conductivities reported for Omc- S and Z, the cell would respectively need to consume $9\times 10^{6}$ and $4\times 10^{9}$ molecules of acetate each second. This calculation is based on the fact that each acetate molecule yields eight electrons, and ∼95% of those are used for ATP synthesis (Bond and Lovley, 2003). Meanwhile, the known respiratory rate of ∼10^6^ e^−^/s only requires ∼10^5^ molecules of acetate per second.

Since the reported conductivities vastly exceed the metabolic needs of a single *G. sulfurreducens* cell, and the cell primarily lives in a solitary state in nature, we take the estimate of the needed conductivity from the respiration rate and consider the solid-state measurements to be biologically irrelevant.

#### 3.6.2 An estimate of maximal single-filament conductivity for cellular respiration

It is not known how many filaments a *G. sulfurreducens* cell expresses, but it is certainly more than one. A study observed >20 filaments thought to be pili (Tan et al., 2016b) but now argued to be OmcS (Wang et al., 2019). A bioenergetic analysis previously suggested that 100 filaments/cell would be reasonable (Levar et al., 2012). If 100 filaments are parallel, carry the entire metabolic flux of electrons, and have the same physical properties of Omc- S or Z, the filaments would need on average a charge diffusion constant by Equation 14 of $7.5\times 10^{-8}$ cm^2^/s.

$$D=\frac{Gk_{b}TL_{w}}{A\rho e^{2}}$$ (14)

Can this diffusion constant be achieved within the above-mentioned energetic bounds for $\sqrt{\langle H^{2}\rangle }$ (T-stacked: 0.002–0.006 eV; slip-stacked: 0.007–0.010 eV), $\Delta G^{\circ }$ (±0.3 eV), and $\lambda^{\text{rxn}}$ (0.24–1.06 eV)?

Monte Carlo simulations answered this question in the affirmative. Sets of *N* $\sqrt{\langle H^{2}\rangle }$ , *N* $\Delta G^{\circ }$ , and *N* $\lambda^{\text{rxn}}$ were generated, where *N* is the number of electron transfer steps. Parameter sets were accepted if the associated diffusion constant was within 10% of the target value. For a CN with the TSTSTS unit cell of OmcS or the TSSTSTS unit cell of OmcZ (excluding the heme that branches from the main conductive chain), $1.0\times 10^{6}$ unique sets were found after $6.4–7.5\times 10^{7}$ sets were attempted, respectively.

**FIGURE 13:**
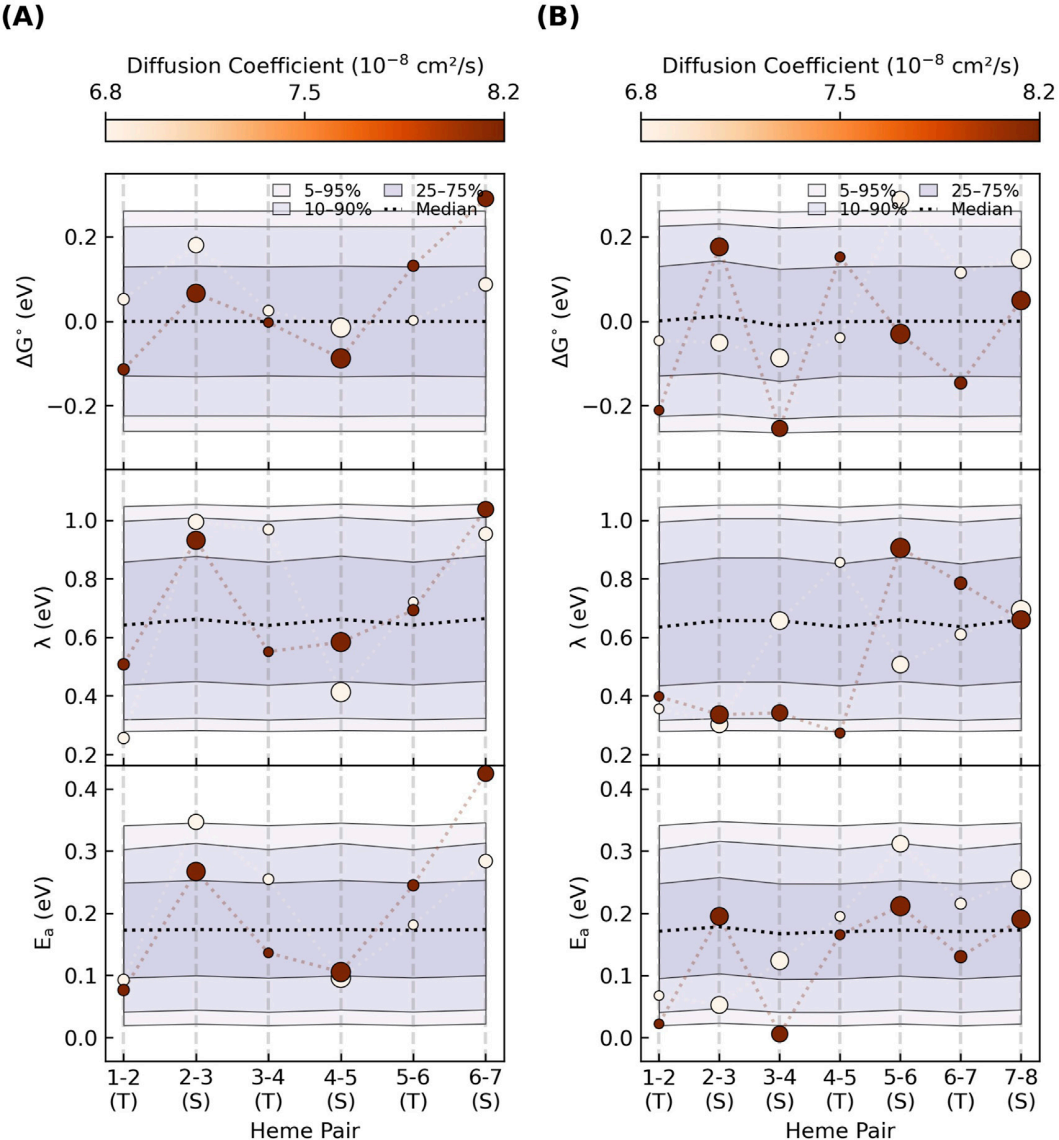
An enormous combination of energetic parameters can yield the maximum single-filament charge diffusion constant needed for cellular respiration. Each panel summarizes the percentile ranges for one-million parameter sets that gave diffusion constants within 10% of $7.5\times 10^{-8}$ cm^2^/s. The markers indicate the sets that gave the highest and lowest diffusion constants, with the size of the markers proportional to the electronic coupling for that step. **(A, B)** show the repeat patterns for heme packing in Omc- S and Z, respectively.

Figure 13 summarizes the statistics for the compatible $\Delta G^{\circ }$ s, $\lambda^{\text{rxn}}$ s, and resulting $E_{a}$ s from the Monte Carlo simulations, as well as the parameter sets that had the largest and smallest diffusion constants within 10% of the target value. Movies of representative parameter sets are included in the Supplementary Material.

#### 3.6.3 Cytochrome “nanowires” suffice for cellular respiration

**FIGURE 14:**
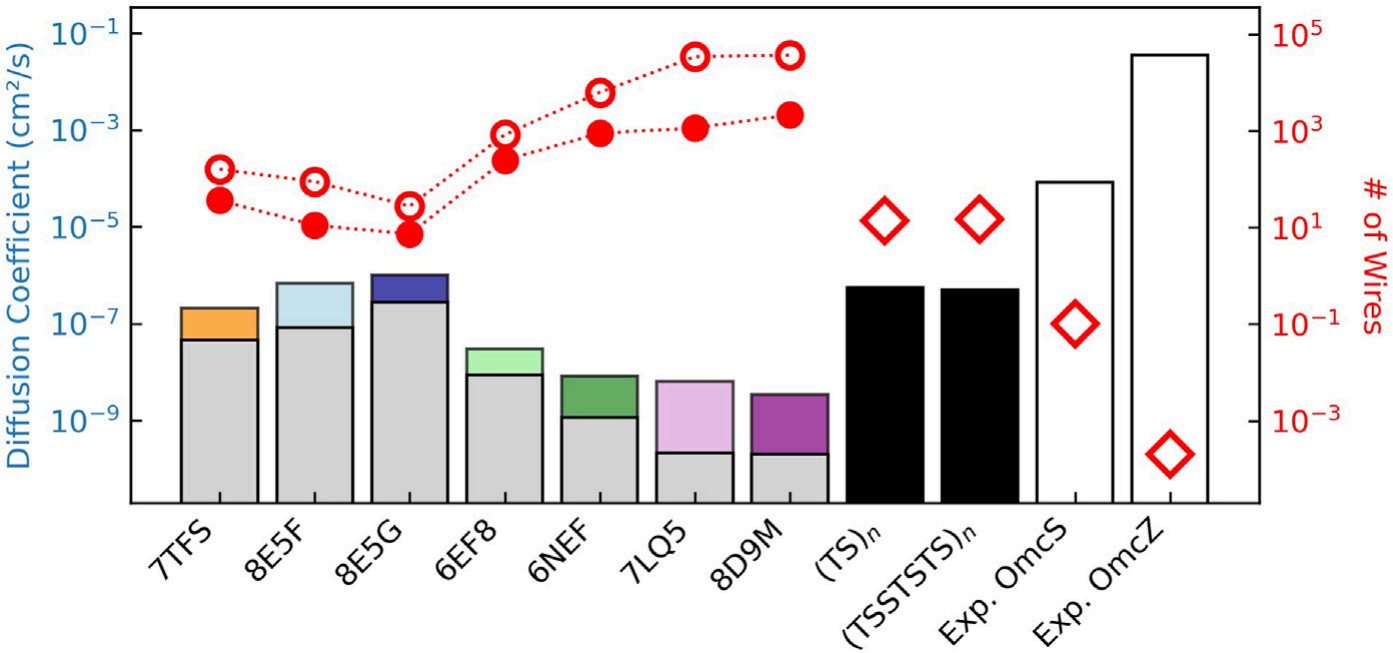
Charge diffusion within structurally characterized cytochrome “nanowires” (colored bars) is sub-optimal compared to heme chains with the same packing geometries but the fastest known electron transfer rates at every step (black bars), which in turn are unphysically dwarfed by the reported conductivities for Omc- S and Z (white bars). The colored versus gray bars show the influence of heme-heme interactions on the free energy landscapes. The red filled/unfilled circles show the number of filaments/cell needed given the computed diffusion constants with/without considering redox anti-cooperativities, and the estimate of the total (cellular) diffusion constant of $7.5\times 10^{-6}$ cm^2^/s needed for parallel filaments. The unfilled diamonds indicate the number of filaments/cell given the diffusion constants obtained for the idealized heme chains or from the reported conductivities.

To make comparisons with the metabolically-required maximal diffusion constant for expelling electrons in the previous section, the set of $k_{\text{et}}$ s for moving an electron through a unit cell of each homopolymeric CN was used to evaluate the analytical Derrida formula (Derrida, 1983) for diffusive charge hopping along the infinitely periodic (pseudo)-one-dimensional heme chains. The obtained diffusion constants and the predicted number of filaments/cell given those values are shown in Figure 14.

Diffusion constants (colored bars) range from $0.8–2.7\times 10^{-7}$ cm^2^/s for the tetrahemes A3MW92, F2KMU8, and OmcE; $1.1–8.9\times 10^{-8}$ cm^2^/s for OmcS depending on the CryoEM structure; and $2.0–2.2\times 10^{-10}$ cm^2^/s for OmcZ depending on the CryoEM structure. These diffusion constants are typically no more than a factor of eight larger than when redox anti-cooperativities are neglected (gray bars) in the geometrically imposed linear sequence of heme oxidations. The exception is OmcZ for which the increase is 17- (PDB 8D9M) or 30-fold (PDB 7LQ5). Differences between the colored and gray bars reflect the influence of redox anti-cooperativities on the $\Delta G^{\circ }$ landscapes, as discussed in Section 3.2.3.

If the entire respiratory flux of electrons was discharged through parallel CNs having these diffusion constants, tens to hundreds of the tetra- or hexahemes, or thousands of the octaheme (red markers in Figure 14; filled/unfilled circles = with/without redox anti-cooperativities) would be needed. Since the cell may discharge less current than assumed here, the filaments would only need to discharge a fraction of it, and tens to hundreds of filaments are expected to be expressed, the prediction of the *maximal* number of filaments seems reasonable.

#### 3.6.4 Cytochrome “nanowires” are not optimized for electrical conductivity

That the conductivity of CNs is biologically sufficient does not mean it is optimal. We already saw that there are physical constraints on the energetics of electron transfer by virtue of the multiheme architecture. Within those constraints, the question is: How ‘good’ are the CNs for redox conduction compared to idealized heme chains with the same sequence of slip- and T-stacked packing motifs, but with the fastest known experimental electron transfer rates ( $k_{\text{et},\text{slip}-\text{stakced}}=1.56\times 10^{9}$ and $k_{\text{et},T-\text{stacked}}=1.25\times 10^{8}$ s^-1^) (van Wonderen et al., 2019; van Wonderen et al., 2021) assigned to each step?

The tetra-, hexa- and octahemes have electron diffusion constants that are respectively one, two, and three orders of magnitude lower than the idealized heme chains (Figure 14, black bars). Those idealized heme chains with maximal conductivities based on 1–10 ns electron transfers would, in turn, have diffusion constants that are two to four orders of magnitude lower than suggested from the reported conductivities for Omc- S and Z (Figure 14, white bars).

**FIGURE 15:**
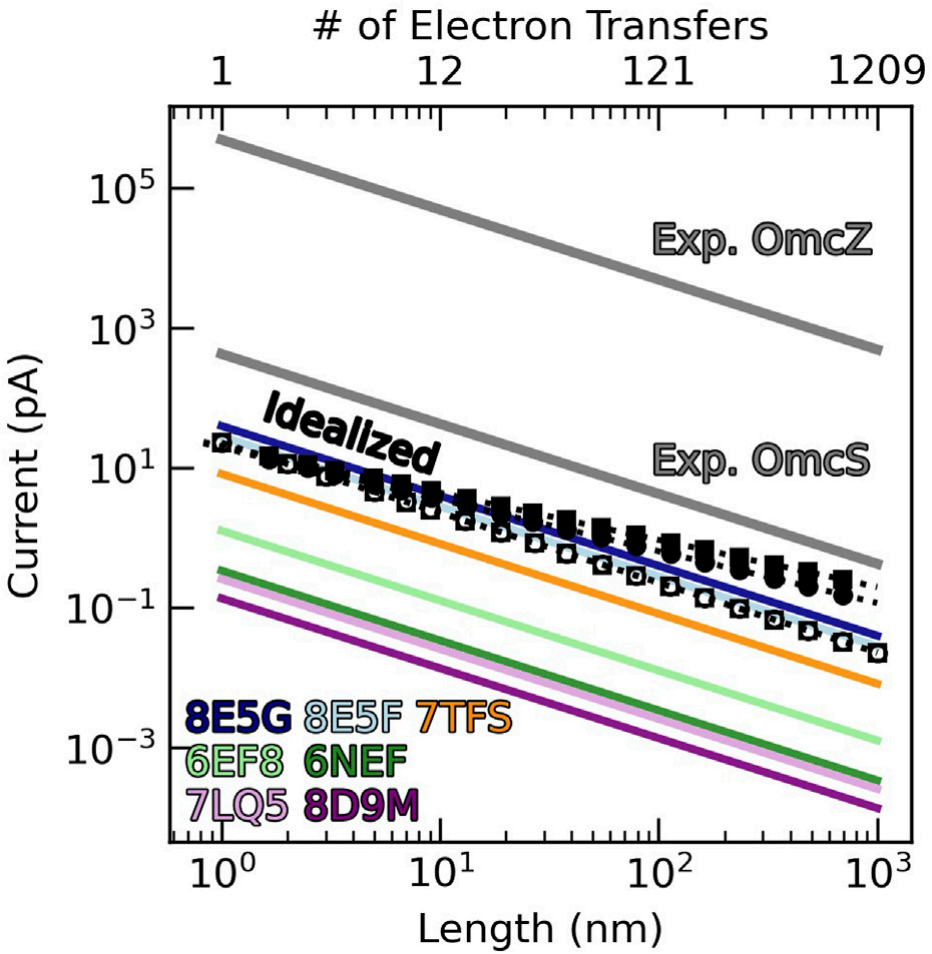
Cytochrome “nanowires” are sub-picoamp conductors. The black unfilled circles and squares show the diffusive current at 0.1 V through the heme chains of Omc- S and Z, but with the fastest experimentally known heme-to-heme electron transfer rate at each step. The black filled circles and squares are the protein-limited steady-state currents for the same idealized Omc- S and Z chains. The sub-optimal diffusive currents at 0.1 V through structurally characterized cytochrome “nanowires” are shown with colored lines. The unphysically-large-for-a-cytochrome reported currents at 0.1 V through Omc- S and Z are shown in gray.

To give a more physically intuitive perspective, Figure 15 shows the length dependent currents that the various CNs could support at a 0.1 V bias with the computed diffusion constants (colored lines) via Equation 15 (Polizzi et al., 2012). The black open circles and squares in the figure represent the diffusive current through the idealized (TS)_n_ and (TSSTSTS)_n_ chains.

$$I=\frac{Ae^{2}\text{ρ}D}{k_{b}\text{TL}}V$$ (15)

The closed circles and squares in Figure 15 represent the maximal protein-limited current computed using a multi-particle steady-state approach. Because the approach is computationally demanding, an equation for a hopping process (Equation 16) (Ing et al., 2018) was fit $\left(R^{2}=0.87–0.92\right)$ to computed fluxes for chains up to 18 steps and then extrapolated for longer filaments.

$$k_{\text{et}}=k_{N}N^{-\eta }$$ (16)

In Equation 16, $k_{N}$ is the hopping rate and N is the number of hopping steps. $k_{N}$ was $1.4–1.5\times 10^{8}$ s^-1^, nicely reflecting the kinetic constraint of the T-stacked pairs present in either chain. η was 0.67–0.74 for the chains.

Both the diffusive and protein-limited kinetic models agree that a micron-long CN emanating from a microbial cell has a sub-picoamp ampacity. A micron-long chain of either the (TS)_n_ or (TSSTSTS)_n_ heme packing pattern and the fastest known T- and slip-stacked rates at every step can carry a diffusive current at 0.1 V of ∼0.02 pA, or a protein-limited current of ∼0.2 pA. Most of the actual CNs carry smaller currents.

There is no structural basis for the report that OmcZ transports $3.0\times 10^{4}$ pA at 0.1 V (Yalcin et al., 2020). This discrepancy may reflect the fact that the measurement was done on a sample with structural characteristics that are now known to disagree with the CryoEM geometry (Guberman-Pfeffer, 2024), perhaps suggesting the presence of impurities or experimental artifacts.

Taken together, the known CNs are *sufficiently* conductive for cellular respiration, but not *maximally* conductive for a multiheme cytochrome. From a physiological perspective, there is no need to be more conductive, because enzymatic turnover during organic matter oxidation in the cytoplasm, the ferrying of electrons across the periplasm to the CNs by a relay team of proteins, and discharge of the electrons to oxide minerals likely place the rate-limitation on cellular respiration compared to intra-filament electron transfers.

With the conserved heme chain in CNs being sufficiently conductive regardless of the protein packaging, the latter can be customized for habitat specific interactions without having to re-invent the electron transfer mechanism. CNs are structurally diversified but functionally conserved because evolution has favored robustness over tunability in their design (Guberman-Pfeffer, 2023b).

## 4 Conclusion

To transport electrons over long distances, nature connects catalytic centers by chains of cofactors. The chain of heme cofactors in a CN must be densely packed to realize weak (≤0.01 eV) electronic couplings for electron transfer. But this dense packing (1) limits the accessible driving forces between adjacent hemes (≤|0.3| eV) by placing them in shared electrostatic microenvironments, (2) introduces strong (≤0.1 eV) redox anti-cooperativities that would accentuate the free energy landscape if it were not for the linear topology of the heme chain, and (3) incurs an entropic penalty that must be offset by tethering the hemes to the polypeptide backbone via thioether linkages.

These linkages physically require T-stacking of hemes for chains longer than three hemes, and in fact, almost every other heme pair in the CNs is of this geometry. T-stacking imposes ∼3-fold smaller electronic couplings and ∼10-fold slower electron transfer rates than slip-stacking. As the slowest step in the cascade of redox reactions through CNs, the T-stacked pairs put a structurally-encoded limit on the electron efflux.

Prior experiments and the herein presented computations on all structurally characterized CNs indicate electron transfers between T-stacked hemes is constrained to the nanosecond timescale. Whether the electron flux is in a diffusive or protein-limited steady-state regime, a micron-long CN has an ampacity of ∼0.02–0.2 pA.

This result implies tens to thousands of filaments would be needed to carry the *entire* respiratory current of the microorganism known to produce three of the so-far characterized five CNs. Since the cell likely produces less current than the 1 pA assumed here, and the CNs would only need to carry a fraction of the total current because there are other routes for expelling electrons, the obtained micro-to-milli-Siemen/cm conductivities physically imposed by their multiheme architectures are sufficient for cellular respiration.

But these conductivities are not optimal compared to idealized heme chains with the fastest known rates for each step. There is simply no need for more speed since the slowest intra-filament electron transfer is still 10^3^-fold faster than the millisecond timescale for the turnover of metabolic enzymes and interfacial protein-mineral electron transfer.

This conclusion puts in stark contrast the unphysical nature of the reported milli-Siemens-to-Siemens conductivities for CNs. These yet-to-be replicated measurements appear structurally implausible, biologically irrelevant, and theoretically inexplicable for a multiheme in physiological conditions.

Over the past decade these electrical measurements were first attributed to *e*-pili and then ascribed to cytochromes. Either way, the comment of Tender and co-workers seems appropriate, “To date [2015], the only results inconsistent with redox conduction [through cytochromes] occurring in *G. sulfurreducens* biofilms are those reported by Malvankar *et al.*, which have been addressed elsewhere [36] and which have not been corroborated by others” (Boyd et al., 2015).

## Data Availability

The original contributions presented in the study are included in the article/Supplementary Material, further inquiries can be directed to the corresponding author.
